# Milk‐Derived Injectable Wound Dressing with Sequential Photoactivatable Antibacterial Property through In Situ Biomineralization

**DOI:** 10.1002/smsc.202500026

**Published:** 2025-06-23

**Authors:** Qinchao Zhu, Xuhao Zhou, Zhidan Wang, Daxi Ren, Tanchen Ren

**Affiliations:** ^1^ Department of Cardiology The Second Affiliated Hospital School of Medicine Institute of Dairy Science College of Animal Sciences Zhejiang University Hangzhou 310009 China; ^2^ State Key Laboratory of Transvascular Implantation Devices Heart Regeneration and Repair Key Laboratory of Zhejiang Province Hangzhou 310009 China; ^3^ College of Animal Sciences Xinjiang Agricultural University Urumqi 830052 China; ^4^ Transvascular Implantation Devices Research Institute Hangzhou 310009 China

**Keywords:** antibacterial activities, biomineralization, casein, silver nanoparticles, wound healing

## Abstract

The emergence of drug‐resistant bacteria due to excessive antibiotic use has drawn increasing attention to inorganic nanoparticles for their broad‐spectrum antibacterial properties. Here, a “green” strategy for the simultaneous in situ synthesis of silver nanoparticles (AgNPs) during the photocrosslinking process of casein hydrogels is described. The in situ photoactivated biomineralization of AgNPs provides noticeable stability and antibacterial activity, with high photothermal effect during a sequential near‐infrared laser activation. The milk‐derived casein is screened out due to its great biomineralization capability and wound healing activity. Casein‐AgNP hydrogel dressing shows low swelling, good mechanical properties, and nice biocompatibility. In animal experiments, casein‐AgNP hydrogel accelerates wound repair and tissue regeneration after bacterial infection by regulating immune response. Our work shows that sequential photoactivation served as a promising strategy for antiinfectious wound treatment, and casein hydrogel stood as a potential candidate for in situ biomineralization.

## Graphical abstract

A milk‐derived casein protein hydrogel wound dressing with sequential photoactivatable antisepsis is developed. The first photoactivation enables rapid in situ hydrogel gelation and AgNP biomineralization; the second photoactivation enhances bactericidal effect by optothermal. The synergistic antibacterial strategy demonstrates remarkable efficacy in significantly accelerating the recovery of infected wounds.

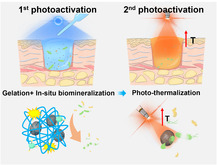

## 1 Introduction

Skin injury commonly occurs in daily life and is susceptible to wound infections. Infections can cause serious complications and hinder skin recovery.^[^ ^1^ ^]^ Coinfections with multiple pathogenic microorganisms have been observed in most infected wounds, as well as the emergence of multidrug‐resistant bacteria, which raises the urgent need for highly efficient antiseptic treatment. Inorganic nanoparticles, notably silver nanoparticles (AgNPs), can interact with various microorganisms (such as bacteria) and impact the growth of mature bacterial biofilms; therefore, they could be used as broad‐spectrum antimicrobials.^[^ ^2^, ^3^ ^]^

In addition to being antibacterial, a wound dressing must act as a partial skin barrier to avoid secondary injury and provide a favorable microenvironment for wound healing.^[^ ^4^, ^5^, ^6^ ^]^ At present, a variety of wound dressings is available for this purpose. Among these, injectable hydrogel dressings possess the advantages of tissue‐like structures, excellent flexibility, shape adjustability, diverse components, and degradability, making them competitive candidates for next‐generation wound dressings.^[^ ^7^, ^8^, ^9^ ^]^ Endowing hydrogel dressing with AgNPs has become an effective strategy for antibacterial wound dressing.^[^ ^10^, ^11^, ^12^, ^13^ ^]^

Conventional methods for mixing AgNPs with pregel polymers have several limitations: i) residuals of potentially toxic agents during the synthesis of AgNPs and ii) the risk of AgNP aggregation and oxidation‐induced loss of efficacy during storage.^[^ ^14^ ^]^ In situ biomineralization is an efficient and eco‐friendly process that facilitates the integration of inorganic materials with organic macromolecules.^[^ ^15^, ^16^ ^]^ In particular, the synthesis of metal nanostructures using biomineralizing agents, such as proteins and other hydrophilic polymers, has attracted considerable interest because of their superior biocompatibility, conformal interfaces, uniform distribution, and high stability.^[^ ^17^, ^18^ ^]^ Strategies, including repetitive freeze‐thawing,^[^ ^19^ ^]^ ultraviolet (UV) irradiation,^[^ ^20^ ^]^ and alkaline conditions,^[^ ^21^ ^]^ have been integrated to promote biomineralization of different proteins for AgNP synthesis. However, the in situ synthesis of AgNP/hydrogels still shows limited biomineralization efficiency. For example, the formation of collagen‐AgNP hydrogels spared a 0.5 h incubation under light exposure.^[^ ^22^ ^]^

The strong reducibility of plant polyphenols has been exploited to improve their biomineralization capacity.^[^ ^23^, ^24^, ^25^ ^]^ Modifying gelatin with catechol groups enhanced its biomineralization capacity for in situ AgNP formation.^[^ ^22^ ^]^ The highly reactive free radicals originating from the homolytic cleavage of the photosensitive precursor can also reduce silver cations to AgNPs. Zaier et al. developed a one‐step photo‐induced method for synthesizing silver@polymer nanoassemblies on a variety of substrates.^[^ ^26^ ^]^ Thus, combining polyphenols and a radical “initiator” accelerates AgNP synthesis in hydrogels.

In addition to small‐molecule excipients, macromolecular backbones also play an important role in the biomineralization process. Fu et al. utilized the tyrosine residues in collagen to reduce Ag^+^ under illumination and formed collagen‐AgNP hydrogels in a single step.^[^ ^27^ ^]^ Thus, we hypothesized that choosing a suitable native macromolecular backbone could improve biomineralization. Milk‐derived proteins have long been known to promote bone mineralization.^[^ ^28^ ^]^ As one of the main milk proteins, bovine casein contains a large proportion of tryptophan, tyrosine, histidine, and proline residues, which endows it with certain reducibility.^[^ ^29^, ^30^ ^]^ However, their ability to synthesize AgNPs has not been explored. Previous studies in our laboratory have shown that milk‐derived casein hydrogels have a great ability to bind metal ions^[^ ^31^ ^]^ and promote wound healing.^[^ ^32^ ^]^ Thus, we explored the biomineralization of casein for AgNP synthesis and its effects on the management of infected wounds.

**Figure 1:**
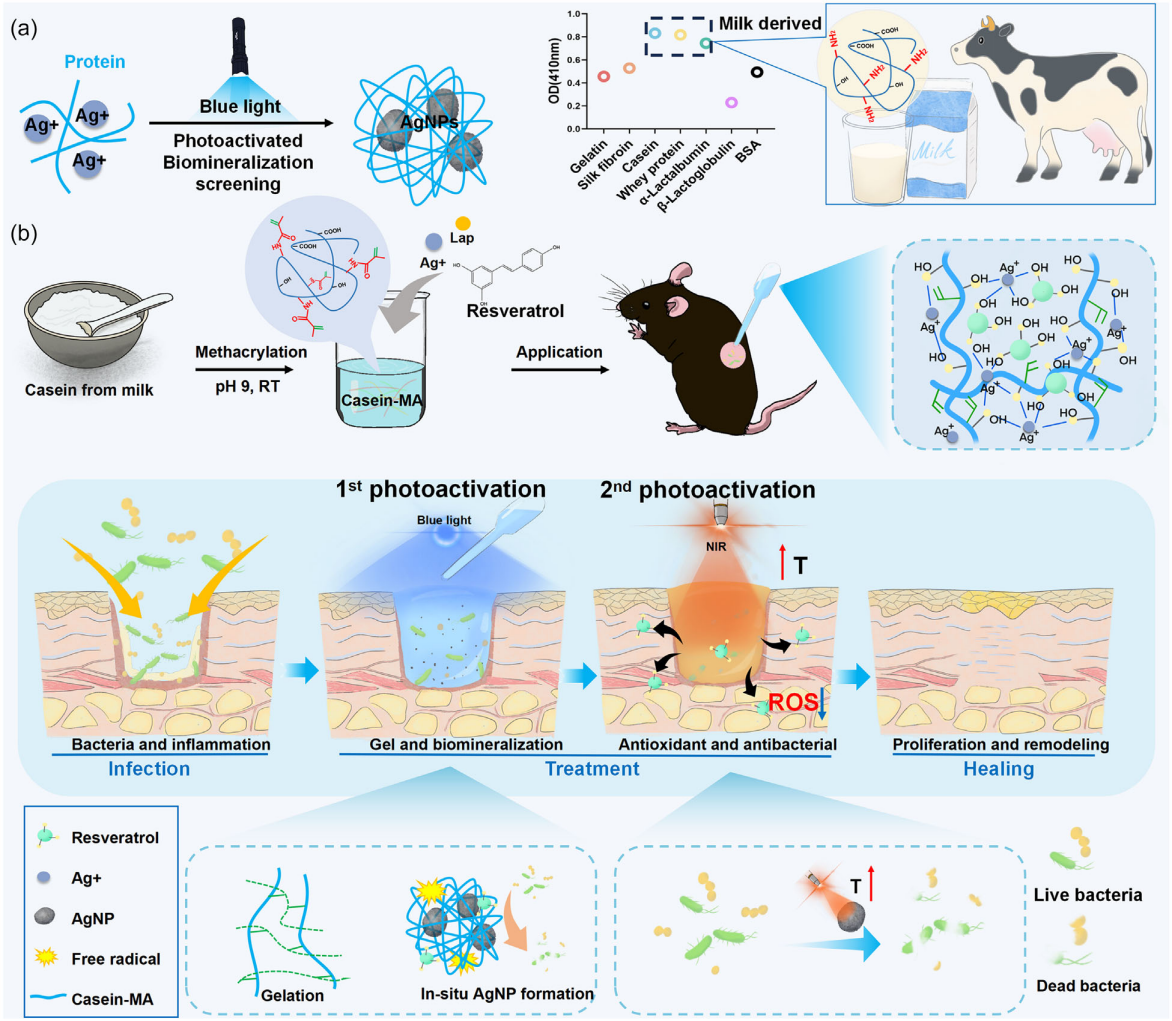
a) Schematic diagram of proteins suitable for photoactivated biomineralization of AgNP. b) Schematic diagram illustrating the sequential photoactivation of CASMA‐Ag hydrogel and its application in infectious wound healing.

In this study, we report an efficient in situ sequential photoactivatable antibacterial hydrogel wound dressing derived from protein biomineralization. To screen suitable proteins, we selected several commonly used protein materials^[^ ^33^, ^34^ ^]^ as candidates and evaluated their photoactivatable biomineralization properties for AgNP generation (**Figure** **1**a). As three milk‐derived proteins (casein, whey protein, and α‐lactalbumin) showed significantly higher AgNP photosynthesis, we ultimately selected casein due to its promising wound healing function, as we showed previously.^[^ ^32^ ^]^ Resveratrol was added to further enhance the mineralization speed for in situ AgNP generation. Casein was methacrylated to enhance the mechanical properties of the hydrogel. Additionally, a photosensitive initiator of free radicals, lithium phenyl‐2,4,6‐trimethylbenzoylphosphinate (LAP), was used to accelerate the AgNP formation.^[^ ^26^ ^]^ The polymerization of methacrylated casein (casein‐MA) and the formation of AgNPs were accomplished simultaneously under the first UV irradiation, which was further amplified by a second near‐infrared (NIR) irradiation (Figure 1b). The sequential photoactivated casein‐AgNP hydrogel (CASMA‐Ag hydrogel) was used as a dressing to treat infectious lesions in mice. The wound tissues were collected for whole genome sequencing to identify the main pathways modulated by the sequentially photoactivated casein‐based hydrogel. Our results suggest that the sequentially photoactivated CASMA‐Ag hydrogel has optimal prospects for application as an antiseptic wound dressing.

## 2 Results and Discussions

### 2.1 Photoactivated Formation of CASMA‐Ag Hydrogel

Biomineralization plays a significant role in wound dressing. Inorganic minerals offer structural reinforcement, support essential cellular processes, and enhance dressing bioactivity owing to their multifunctional properties.^[^ ^35^, ^36^ ^]^ For infected wounds, infection control is a paramount factor influencing healing outcomes. AgNPs are distinguished from other minerals owing to their excellent broad‐spectrum antibacterial properties.^[^ ^37^ ^]^ Additionally, AgNPs with an appropriate particle size range exhibit good photothermal effects, which can facilitate infection clearance and accelerate healing through photothermal therapy.^[^ ^38^ ^]^

**Figure 2:**
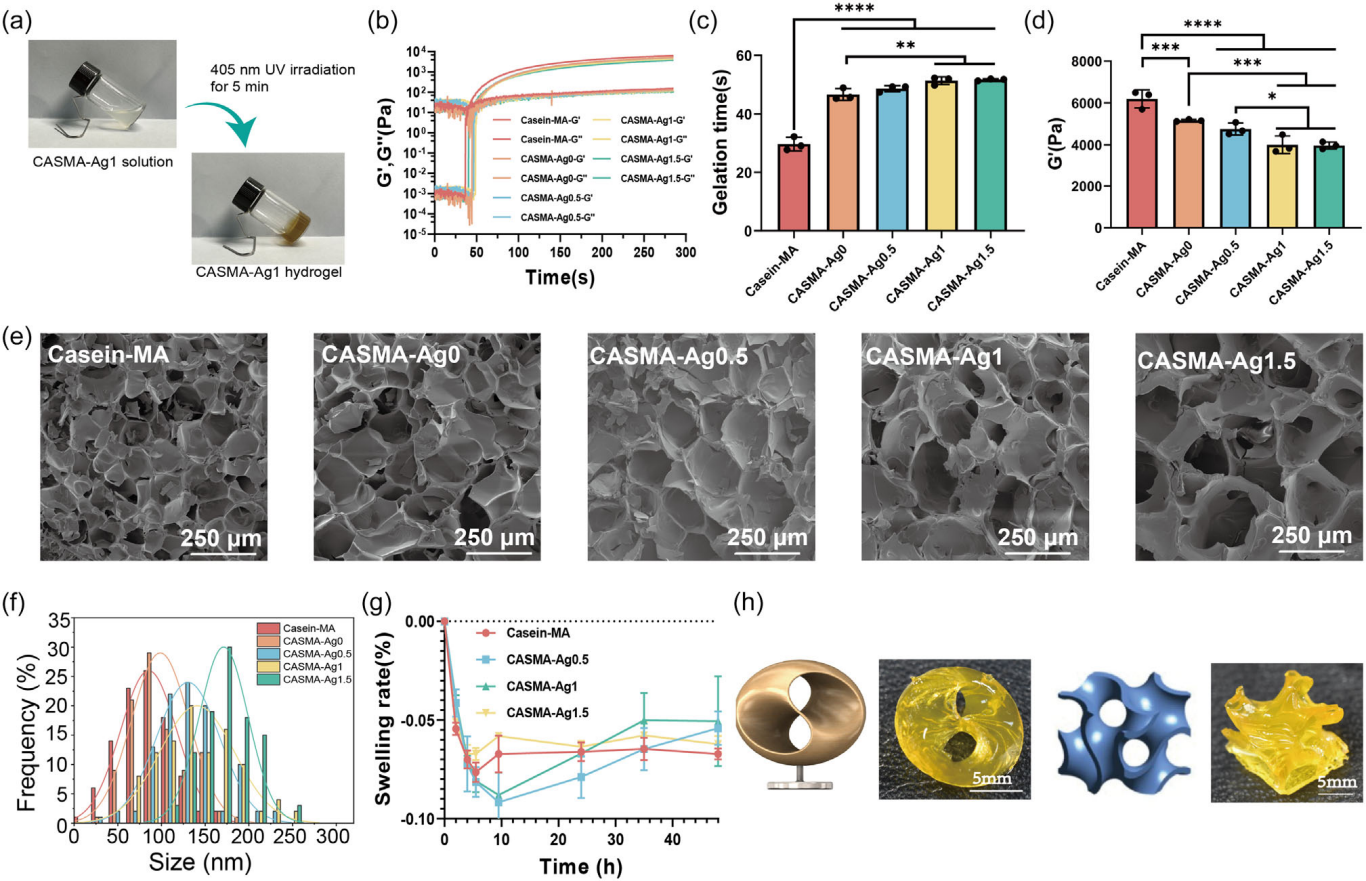
UV‐induced formation of CASMA‐Ag hydrogel. a) Photograph showing the sol‐gel transition of CASMA‐Ag1 upon 405 nm UV irradiation for 5 min. b) Rheology curve. c) Gelation time and d) storage modulus of hydrogels exposed to 405 nm UV irradiation for 300 s (mean ± SD; *P < 0.05, ***P < 0.001, and ****P < 0.0001; n = 3). e) SEM images and f) pore size distribution of different hydrogels. g) Mass swelling rate of hydrogels in PBS within 48 h (n = 3). h) 3D printing of 10% w/v casein‐MA hydrogel.

The biomineralization of different proteins was screened using a photoactivation method. Compared to other native proteins commonly used in biomaterials, casein, whey, and lactoglobulin, the three main components of milk showed the highest biomineralization (Figure 1). To prepare a hydrogel dressing with optimized in situ gelation, biomineralization, and peroxidation, casein was chosen because we have previously shown its function in wound repair.^[^ ^32^ ^]^ To further promote cross‐linking, casein was methacrylated, which did not impair AgNP formation (Figure S1, Supporting Information). Macroscopic images of the CASMA‐Ag1 hydrogel demonstrated the transformation from a colorless and transparent solution into a brown solid hydrogel after exposure to 405 nm irradiation for 5 min (**Figure** **2**a).

The gelation process of casein‐MAa under UV curing was monitored by rheology to assess the kinetics of the photocrosslinking reaction and the effect of biomineralization on crosslinking behavior (Figure 2b,d). A certain concentration of casein‐MA was required to initiate gelation (Figure S2, Supporting Information). Increasing casein‐MA concentration from 10% to 15% reduced the gelation time from 29.73 ± 2.23 s to 18.57 ± 0.48 s and increased the final storage modulus from 6.19 ± 0.43 to 12.38 ± 0.84 kPa. To meet the flexibility requirements of wound dressings, we used 10% casein‐MA in the following experiments unless otherwise specified.

The addition of resveratrol increased the gelation time by 55% and decreased the modulus to 5.2 kPa. Gelation was further retarded by increasing [Ag^+^]. However, the gelation time was still within 1 min, and the storage modulus was >4.0 kPa for all conditions. The negative influence on the gelation of resveratrol and Ag^+^ may have occurred because of their free radical scavenging properties; resveratrol is well‐known as a free radical quencher,^[^ ^39^ ^]^ and Ag^+^ can react with radicals generated from LAP to form AgNPs.^[^ ^26^ ^]^

The hydrogel microstructures were observed using scanning electron microscopy (SEM). Figure 2e,f shows that all hydrogels had a porous structure, with the addition of resveratrol and Ag^+^ having larger pore sizes.

Hydrogels tend to swell in various aqueous solutions, such as wound exudates, which not only reduces their mechanical properties but also causes adverse compression of the surrounding tissues in vivo.^[^ ^40^ ^]^ The swelling ratios were measured at different time points after soaking the samples in phosphate buffered solution (PBS) for 48 h (Figure 2g). All hydrogels (casein‐MA, CASMA‐Ag0.5, CASMA‐Ag1, and CASMA‐Ag1.5) showed no obvious swelling during the soaking process, which is consistent with our previous swelling results for casein hydrogels crosslinked by the Ru/SPS initiator.^[^ ^32^ ^]^

Owing to its fast gelation, stable structure, and resistance to swelling, photocrosslinkable casein‐MAa is suitable for 3D printing to build a reliable complex architecture. As shown in Figure 2h, the 10% casein‐MA hydrogel precursor solution was successfully cross‐linked layer‐by‐layer into a stereo model, showing good shape fidelity.

### 2.2 Optimization of In Situ Photoactivated Biomineralization and Photothermal Effect

**Figure 3:**
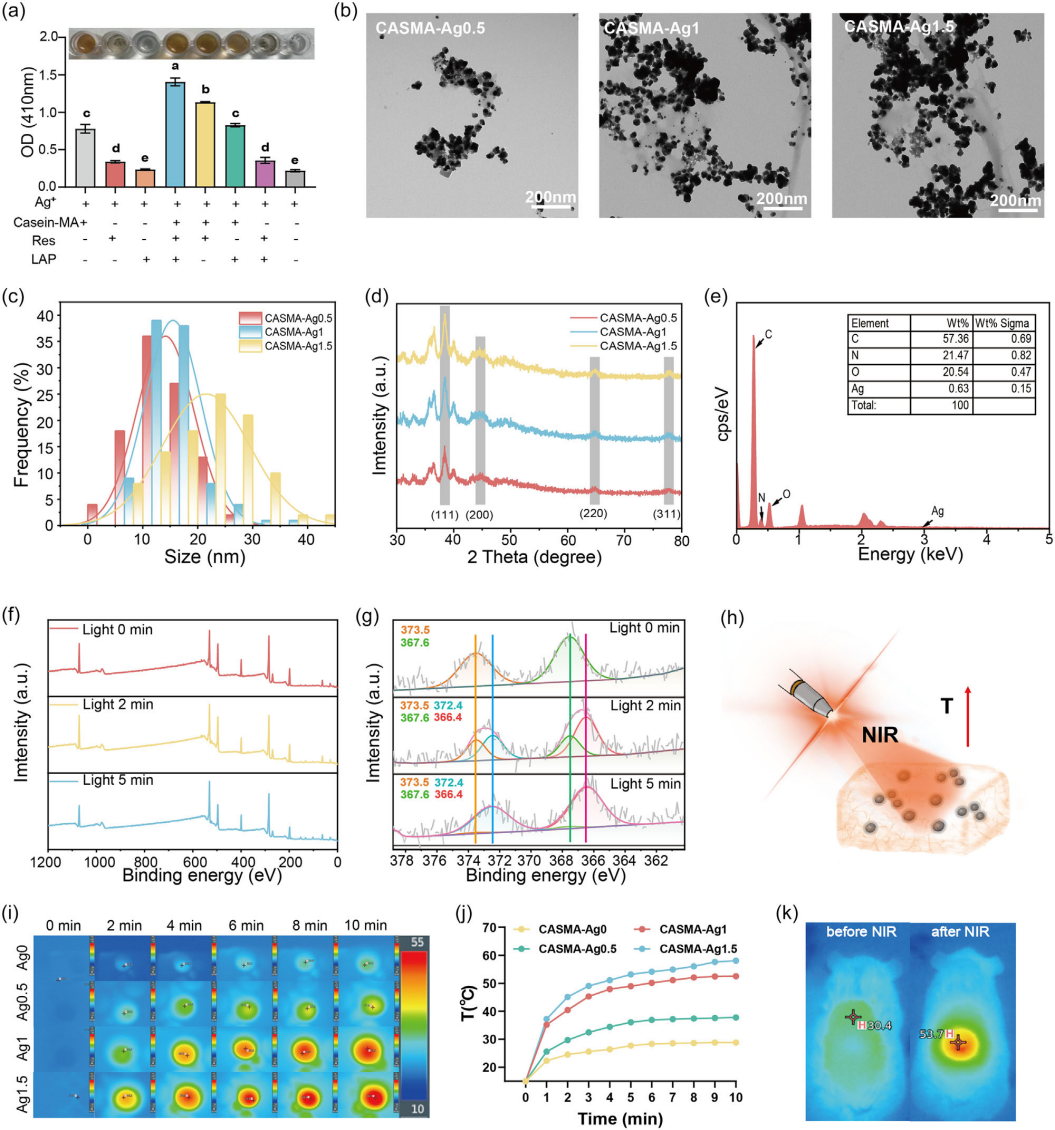
Biomineralization of AgNPs during initial photoactivation and the photothermal effect activated by subsequent NIR irradiation. a) AgNPs generation (OD value at 410 nm) in the presence of different components in the system (mean ± SD; *P* < 0.05; n = 3). b) TEM images of AgNPs in different hydrogels. c) PSD of AgNPs in different hydrogels. d) XRD patterns of AgNPs. e) EDS showing element composition of CASMA‐Ag1. f) Wide‐scan XPS spectra of hydrogels. g) High‐resolution XPS spectra of Ag 3*d*. h) Schematic diagram of the photothermal conversion mechanism in CASMA‐Ag hydrogel. i) Photothermal images showing temperature distribution of different hydrogels under NIR irradiation (3 W cm^−2^, 10 min); j) Corresponding temperature change curves from (i). k) Thermal imaging of a mouse treated with CASMA‐Ag1 hydrogel before and after NIR irradiation (3 W cm^−2^, 10 min).

The kinetics of biomineralization were studied by tracing the light exposure time of AgNP formation (Figure S3, Supporting Information). The peak strength at ≈410 nm increased with light exposure time until it reached a plateau, indicating complete mineralization of the AgNPs. The color shift of the hydrogel during light irradiation indicated the formation of AgNPs. We analyzed the contribution of each component to biomineralization in a mixture of pregel solutions by depleting certain constituents. The photoinitiator LAP and polyphenol resveratrol in the system triggered AgNP synthesis, but the major effect was caused by casein‐MA (**Figure** **3**a). In addition, we observed the agglomeration of silver during photoactivation in groups without casein‐MA, indicating its role as a stabilizer and dispersant for AgNP synthesis. The amount of resveratrol also affected the rate of AgNP synthesis (Figure S4, Supporting Information). However, owing to the limited solubility of resveratrol, highly concentrated resveratrol did not significantly increase the synthesis rate of AgNPs.

To directly observe the morphology of AgNPs formed during photoactivated biomineralization, a transmission electron microscope (TEM) was used. AgNPs were clearly formed by photoactivation, and the amount and size of the AgNPs positively correlated with the initial [Ag^+^] (Figure 3b). The sizes of the AgNPs were 2.5−25 nm for CASMA‐Ag0.5, 5−35 nm for CASMA‐Ag1, and 2.5−45 nm for CASMA‐Ag1.5 (Figure 3c). The particle sizes of the AgNPs in different hydrogels had a broad distribution range. AgNPs in CASMA‐Ag0.5, CASMA‐Ag1, and CASMA‐Ag1.5 present a z‐average diameter of 10.7 nm, 14.4 nm, and 19.2 nm, respectively, which was consistent with the TEM results (Figure S5, Supporting Information). To investigate the impact of polymer concentration on the nanostructures, we increased the concentration of casein‐MAa to 15% w/v for the biomineralization reaction. The morphology of the AgNPs in the 15% casein‐MA system resembled a cubic shape, indicating that the growth direction of the AgNPs was restricted in highly concentrated solutions (Figure S6, Supporting Information).^[^ ^41^ ^]^The results further supported that a casein‐MA concentration of 10% w/v is more suitable for the in situ mineralization of AgNPs.

The X‐ray diffraction (XRD) patterns of CASMA‐Ag0.5, CASMA‐Ag1, and CASMA‐Ag1.5 in Figure 3d show four distinct diffraction peaks at 38.12°, 44.30°, 64.45°, and 77.41°, which correspond to the (111), (200), (220), and (311) crystal faces of the AgNPs, respectively. The results indicated the generation of metallic Ag with cubic face‐centered symmetry, confirming the successful generation of AgNPs in the hydrogels.

The elemental composition of the CASMA‐Ag1 hydrogels was investigated using energy‐dispersive spectroscopy (EDS). The EDS results indicate the presence of Ag (Figure 3e). The uniform distributions of C, O, N, and Ag in the CASMA‐Ag1 hydrogels are shown in Figure S7, Supporting Information. The broad X‐ray photoelectron spectroscopy (XPS) results in Figure 3f do not show any differences in CASMA‐Ag1 after irradiation for 0, 2, and 5 min. However, the fine scan of Ag 3*d* (Figure 3g) showed variation with irradiation duration. Considering the standard spectra from National Institute of Standards and Technology, the binding energy of Ag^+^ was higher than that of Ag^0^. The peaks at 373.5 eV and 367.6 eV were assigned to Ag^+^, while those at 372.4 eV and 366.4 eV were assigned to AgNPs. The AgNPs/Ag^+^ ratio was 1.8 after 2 min of irradiation, and the AgNPs/Ag^+^ ratio increased to 25.6 after 5 min of irradiation. The AgNP peaks became stronger, and the AgNP/Ag^+^ ratio increased with longer photoreduction times. Several studies^[^ ^3^, ^42^, ^43^ ^]^ show AgNPs adhere to and accumulate on bacterial surfaces, disrupt cell membranes, and cause structural changes. Moreover, when nanoparticles dissolve in water or enter cells, a certain amount of Ag^+^ is released, which also exhibits antibacterial function.

Subsequently, the photothermal performance of the CASMA‐Ag hydrogel was studied. The AgNPs exhibited high absorption of NIR light, which could be effectively converted into heat (Figure 3h).^[^ ^44^ ^]^ Figure 3i,j shows that the temperature of the CASMA‐Ag0 hydrogel did not increase significantly under NIR irradiation (3 W cm^−2^). However, after the in situ biomineralization and synthesis of AgNPs, the photothermal response increased significantly. The temperature difference (ΔTS) of CASMA‐Ag0.5, CASMA‐Ag1, and CASMA‐Ag1.5 hydrogels before and after 10 min irradiation reached 22.9, 37.6, and 43.1 °C, respectively, indicating that the introduction of AgNPs provided satisfactory photothermal properties to the hydrogels. Furthermore, the CASMA‐Ag1 hydrogel demonstrated exceptional photothermal stability despite three cycles of on–off NIR radiation (Figure S8, Supporting Information). The maximum heating temperature did not show an obvious decrease, and the ΔTs increased to 36.7, 38.4, and 41.8 °C, respectively. For the CASMA‐Ag1 group, the regional temperature of the mouse increased from 30.4 to 53.7 °C under NIR for 10 min (Figure 3k). These results suggest that the designed CASMA‐Ag hydrogel exhibits excellent NIR photothermal performance, which could aid in the treatment of bacteria‐infected wounds.

The stability of the AgNPs in the CASMA‐Ag1 hydrogel was assessed by tracing the characteristic peaks and morphology over time using UV–vis spectroscopy and TEM. After 20 days of aging time at the storage temperatures of 37 °C, the UV–vis spectrum of the hydrogel and morphology of AgNPs remained unchanged (Figure S9, Supporting Information), indicating the stability of the AgNPs within the hydrogel.

### 2.3 Biocompatibility of CASMA‐Ag Hydrogels

**Figure 4:**
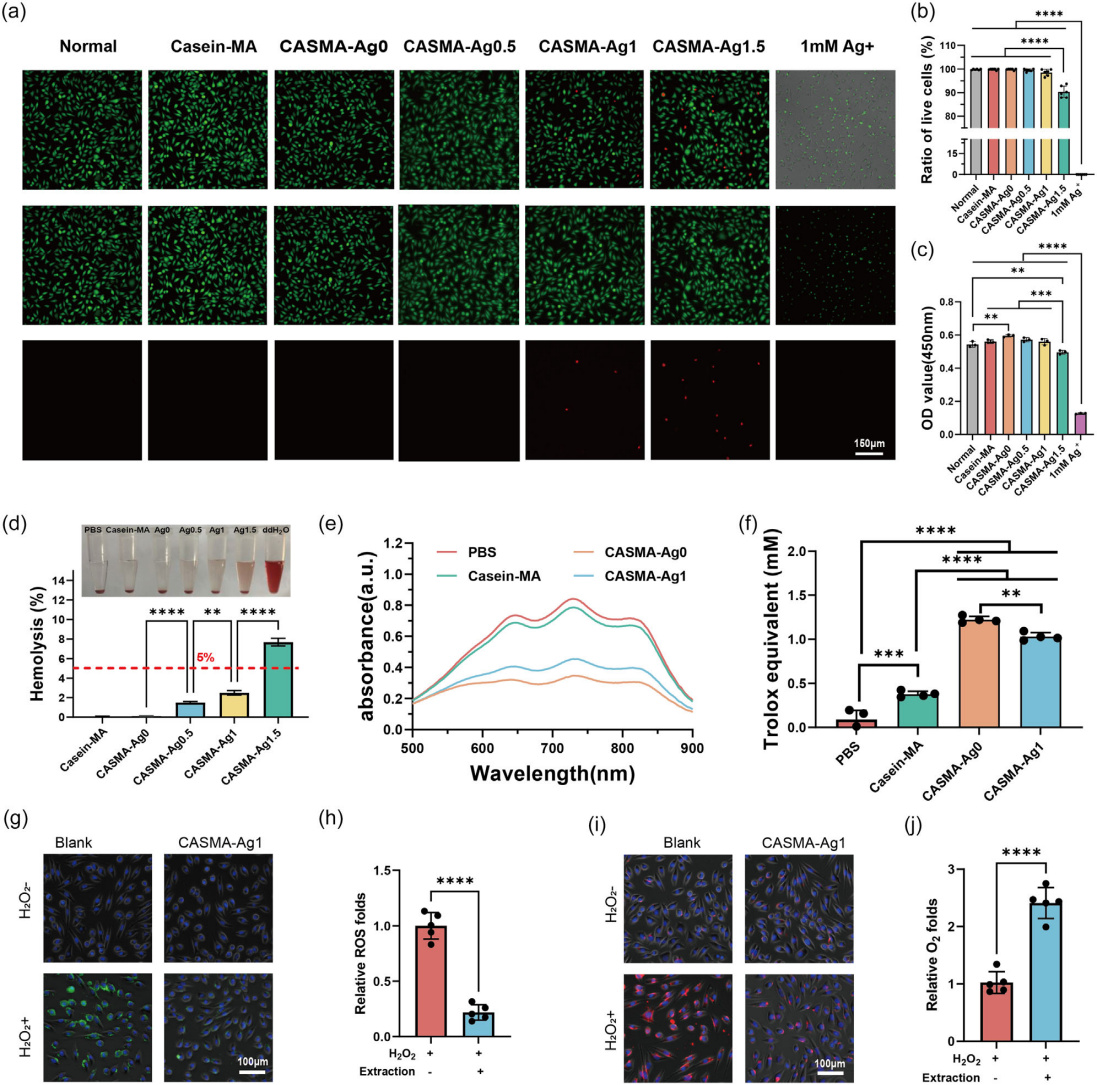
Biocompatible and antioxidant activity of CASMA‐Ag hydrogels. a) Fluorescence microscopy images of live/dead staining of L929 with different treatments. b) Quantification of live cell ratio (mean ± SD; ****P < 0.0001; n = 5). c) Cell viability assessed by CCK‐8 assay (OD at 450 nm) in treated cells (mean ± SD; **P < 0.01, ***P < 0.001, and ****P < 0.0001; n = 3). d) Hemolysis ratios of erythrocytes treated with different hydrogel extracts (mean ± SD; **P < 0.01, and ****P < 0.0001; n = 3). e,f) ABTS + scavenging activity of different hydrogels (mean ± SD; **P < 0.01, ***P < 0.001, and ****P < 0.0001; n = 4). g) ROS levels in L929 cells detected using the DCFH‐DA probe after exposure to H_2_O_2_ and hydrogel extracts. h) Quantitative assay of intracellular ROS depletion based on fluorescence intensity in L929 cells (mean ± SD; ****P < 0.0001; n = 5). i) O_2_ generation in L929 cells detected via Ru(dpp)_3_Cl_2_ probe following H_2_O_2_ and hydrogels. j) Quantitative assay of intracellular O_2_ depletion by measuring the fluorescence intensity in L929 cells (mean ± SD; ****P < 0.0001; n = 5).

Biocompatibility is a critical prerequisite for the applications of materials in biomedicine.^[^ ^45^ ^]^ Hence, cytocompatibility and hemocompatibility experiments were performed to evaluate the biocompatibility of the CASMA‐Ag hydrogels. As shown in **Figure** **4**a,b, casein‐MA, CASMA‐Ag0, CASMA‐Ag0.5, and CASMA‐Ag1 had high cytocompatibility, as cell viability was similar to that of the normal culture. However, free Ag^+^ at a concentration of 1 mM killed almost all cells in the system. Notably, the optical density (OD) values of the CASMA‐Ag hydrogel (CASMA‐Ag0, CASMA‐Ag0.5, and CASMA‐Ag1) groups were slightly higher than those of the casein‐MA group in the Cell Counting Kit‐8 (CCK8) experiment (Figure 4c). This may have been caused by the residual resveratrol, which induces nicotinamide adenine dinucleotide (NADH) oxidation. As the main component of mitochondrial respiration, NADH can reduce the water soluble tetrazolium salt WST‐8 to a water soluble dye (formazan) through dehydrogenase in the mitochondria, which increases absorption at 450 nm.^[^ ^46^ ^]^ Thus, the cytotoxicity decreased significantly after Ag^+^ was mineralized in situ to form AgNPs, which were successfully embedded in the casein hydrogel skeleton, further enhancing biosafety. In addition, increasing the initial [Ag^+^] to 1.5 mM for AgNP formation (CASMA‐Ag1.5 group) significantly lowered cell viability; thus, we used CASMA‐Ag1 for subsequent wound treatment experiments.

Hemocompatibility is an important parameter for biomaterials; therefore, we also tested the hemolytic properties of hydrogels The hemolysis ratios of CASMA‐Ag0, CASMA‐Ag0.5, and CASMA‐Ag1, and CASMA‐Ag1.5 were 0.11 ± 0.02%, 1.49 ± 0.13%, 2.50 ± 0.23%, and 7.69 ± 0.39%, indicating that hemolysis rates slightly increased with increased Ag content. Overall, except for CASMA‐Ag1.5, the hydrogels exhibited satisfactory hemolysis performance (<5%), with negligible damage to erythrocytes (Figure 4d).^[^ ^47^ ^]^ Based on the cytotoxicity and hemolytic tests, CASMA‐Ag1 was used for subsequent experiments.

In vivo biocompatibility was evaluated by subcutaneous implantation in mice. We observed that the hydrogel matrix underwent gradual degradation owing to cellular infiltration overtime, with nearly complete degradation achieved at 28 d (Figure S10, Supporting Information). Hematoxylin and eosin (H&E) staining showed no significant pathological alterations in the major internal organs, including the hearts, livers, spleens, lungs, and kidneys, of mice with CASMA‐Ag1 implantation, compared to the control group (Figure S11A, Supporting Information). Blood biochemical analyses indicated that liver and kidney functions were not affected by CASMA‐Ag1 implantation (Figure S11B, Supporting Information). In summary, the CASMA‐Ag1 hydrogel exhibits negligible systemic toxicity, confirming its outstanding biocompatibility.

### 2.4 Antioxidant Activity of CASMA‐Ag1 Hydrogel

Overproduction of reactive oxygen species (ROS) during the progression of an infected wound microenvironment seriously impairs wound healing.^[^ ^48^ ^]^ Therefore, wound dressings with antioxidant activity have a positive effect on the healing of inflammatory wounds. In theory, resveratrol built into the CASMA‐Ag1 hydrogel can scavenge free ROS and maintain intracellular metabolism.^[^ ^39^, ^49^, ^50^, ^51^, ^52^ ^]^ To evaluate the antioxidant activity of hydrogels, the scavenging efficiency of the 2,2’‐azinobis (3‐ethylbenzthiazolin‐6‐sulfonic acid) (ABTS+) stable radical cation was examined.^[^ ^53^ ^]^ Figure 4e,f shows that, compared with the PBS group, the pure casein‐MA hydrogel had a certain antioxidant activity, which is consistent with previous work.^[^ ^54^ ^]^ The antioxidant activities of CASMA‐Ag0 and CASMA‐Ag1 were significantly higher; therefore, the addition of resveratrol enhanced the antioxidant ability. With the addition of Ag^+^, the antioxidant activity of the hydrogels decreased, indicating that the in situ synthesis of AgNPs dampened the antioxidant activity of the hydrogels. In conclusion, the CASMA‐Ag1 hydrogel exhibited excellent antioxidant activity.

We further evaluated the ROS scavenging effect of the CASMA‐Ag1 hydrogel by treating L929 fibroblasts with H_2_O_2_ to simulate an oxidative microenvironment.^[^ ^55^ ^]^ Intracellular ROS levels were detected using a ROS probe, 2′,7′‐dichlorodihydrofluorescein diacetate (DCFH‐DA). L929 cells coincubated with the CASMA‐Ag1 hydrogel emitted significantly lower green fluorescence than the control group, indicating its profound ROS scavenging ability (Figure 4g,h). Intracellular changes in [O_2_] were measured using the indicator Ru(dpp)_3_Cl_2_. As depicted in Figure 4i−j, the intracellular fluorescence of cells treated with the CASMA‐Ag1 hydrogel was significantly lower than that of cells from the control groups (p < 0.001), indicating that [O_2_] from cells treated with CASMA‐Ag1 hydrogels was significantly higher than that of cells in the control groups. Research suggests that the effect of resveratrol on H_2_O_2_‐mediated death signaling could be attributed to (i) its ability to maintain a higher intracellular O_2_ concentration and (ii) its ability to block H_2_O_2_‐induced cytosolic acidification, thereby creating an environment that is nonpermissive for caspase activation and efficient cell death.^[^ ^56^ ^]^ The addition of resveratrol not only enhanced the biomineralizing effect of hydrogels but also increased hydrogel cytocompatibility and improved the microenvironment of inflammatory wounds.

### 2.5 Antibacterial Properties of CASMA‐Ag Hydrogels

**Figure 5:**
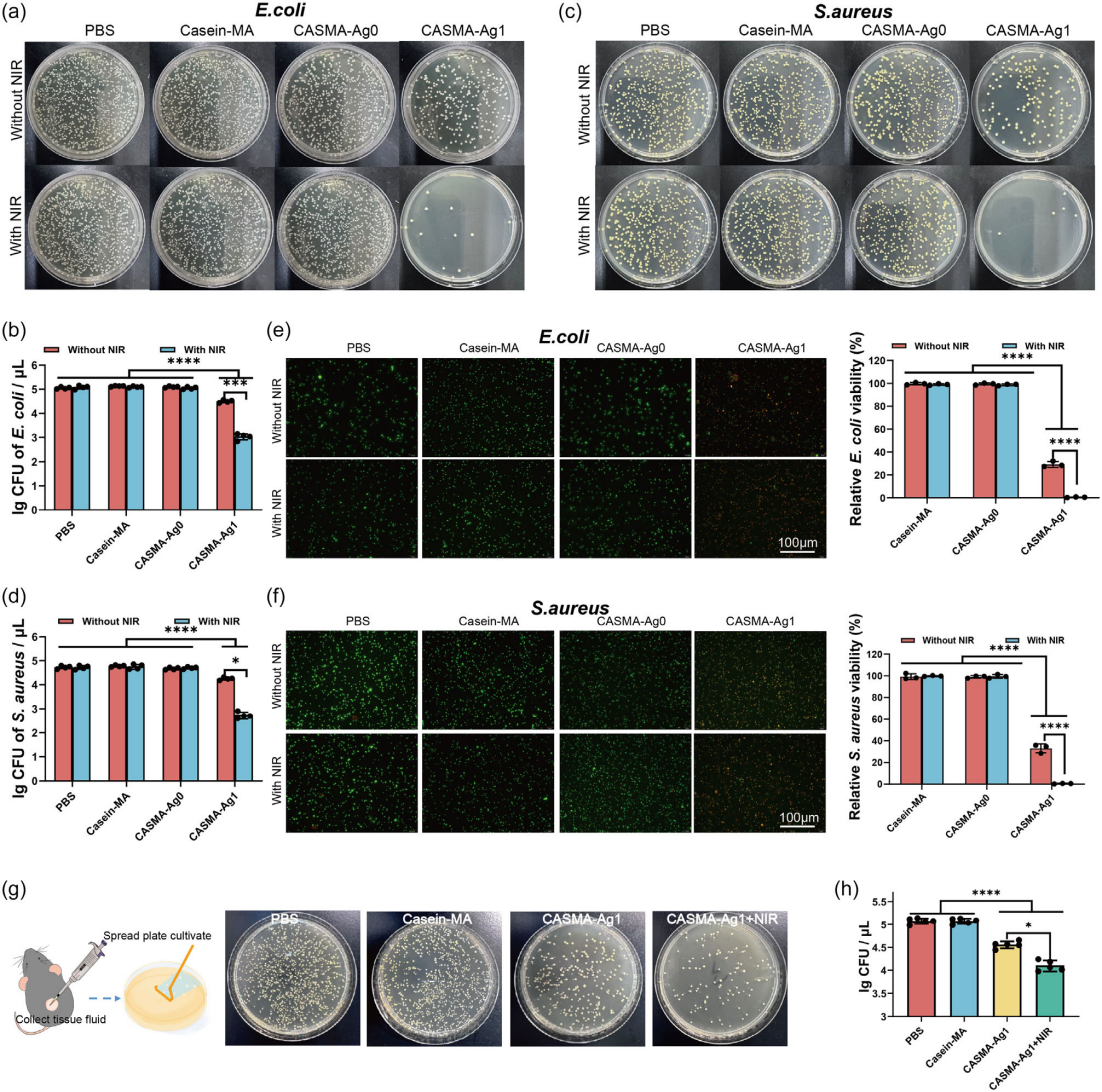
Synergistic antimicrobial efficacy of CASMA‐Ag hydrogels following sequential photoactivation. a,c) Photographs of *E. coli* and *S. aureus* colonies after different treatments with or without NIR irradiation (3 W cm^−2^, 10 min). b,d) Quantification of bacterial CFUs of *E. coli* and *S. aureus* after different treatments with or without NIR (mean ± SD; **P < 0.01, ***P < 0.001, ****P < 0.0001; n = 4). e,f) Fluorescence images of live/dead staining of *E. coli* and *S. aureus* after different hydrogel treatment and their corresponding quantitative analysis. Green represents live bacteria, and red represents dead bacteria. (mean ± SD; ****P < 0.0001; n = 3) g) Schematic of *S. aureus* collection from wound exudates, along with representative images of colonies cultured for 48 h following different treatments. h) Quantitative CFU analysis from (g) (mean ± SD; **P < 0.01, ****P < 0.0001; n = 5).

The spread plate method was used to assess the antimicrobial performance of the hydrogels against gram‐negative (*Escherchia coli*) and gram‐positive (*Staphylococcus aureus*) bacteria, which are responsible for most infections. As presented in **Figure** **5**a−d, in the PBS group, the total number of bacterial colonies was the same with or without NIR, indicating that the 3 W 808 nm laser did not affect the growth of *E. coli* or *S. aureus*. On casein‐MA and CASMA‐Ag0 hydrogel treatments with or without NIR irradiation, the total number of bacterial colonies was similar.

CASMA‐Ag1 hydrogel decreased the number of viable bacteria of *S. aureus* and *E. coli* by 0.5 orders of magnitude compared to AgNP‐free groups after 24 h incubation. After NIR irradiation (3 W cm^−2^, 10 min), the photothermal CASMA‐Ag1 hydrogel reduced the number of viable *S. aureus* and *E. coli* by 1.98 and 2.04 orders of magnitude, respectively. The CASMA‐Ag1 injectable hydrogel, thus, had strong antibacterial properties under the synergistic effect of sequential UV and NIR photoactivation.

To further explore the antibacterial properties of the CASMA‐Ag1 hydrogel, live/dead bacteria fluorescence staining by SYTO9/propidiun iodide (PI) was performed. All live and dead bacteria were stained with SYTO9 with green fluorescence, whereas red fluorescent (PI) staining revealed membrane‐damaged bacteria. As shown in Figure 5e, control, casein‐MA, and CASMA‐Ag0, with or without NIR irradiation, were dominated by green fluorescence and barely showed red fluorescence. In contrast, red fluorescence was observed in the CASMA‐Ag1 hydrogel‐treated group, which increased steadily with NIR treatment. When treated with the CASMA‐Ag1 hydrogel, the viability of the bacterial cells was significantly reduced to < 33% and < 29% for *S. aureus* and *E. coli*, respectively (Figure 5f). Furthermore, CASMA‐Ag1 + NIR treatment resulted in a > 99% reduction in the viability of both *S. aureus* and *E. coli*. The results demonstrated the bactericidal effect of the sequentially photoactivated CASMA‐Ag1 hydrogel and the microbicidal effect of each photoactivation step.

### 2.6 In Vivo Wound Healing of the CASMA‐Ag1 Hydrogel in a Mouse Full‐Thickness Wound Model

**Figure 6:**
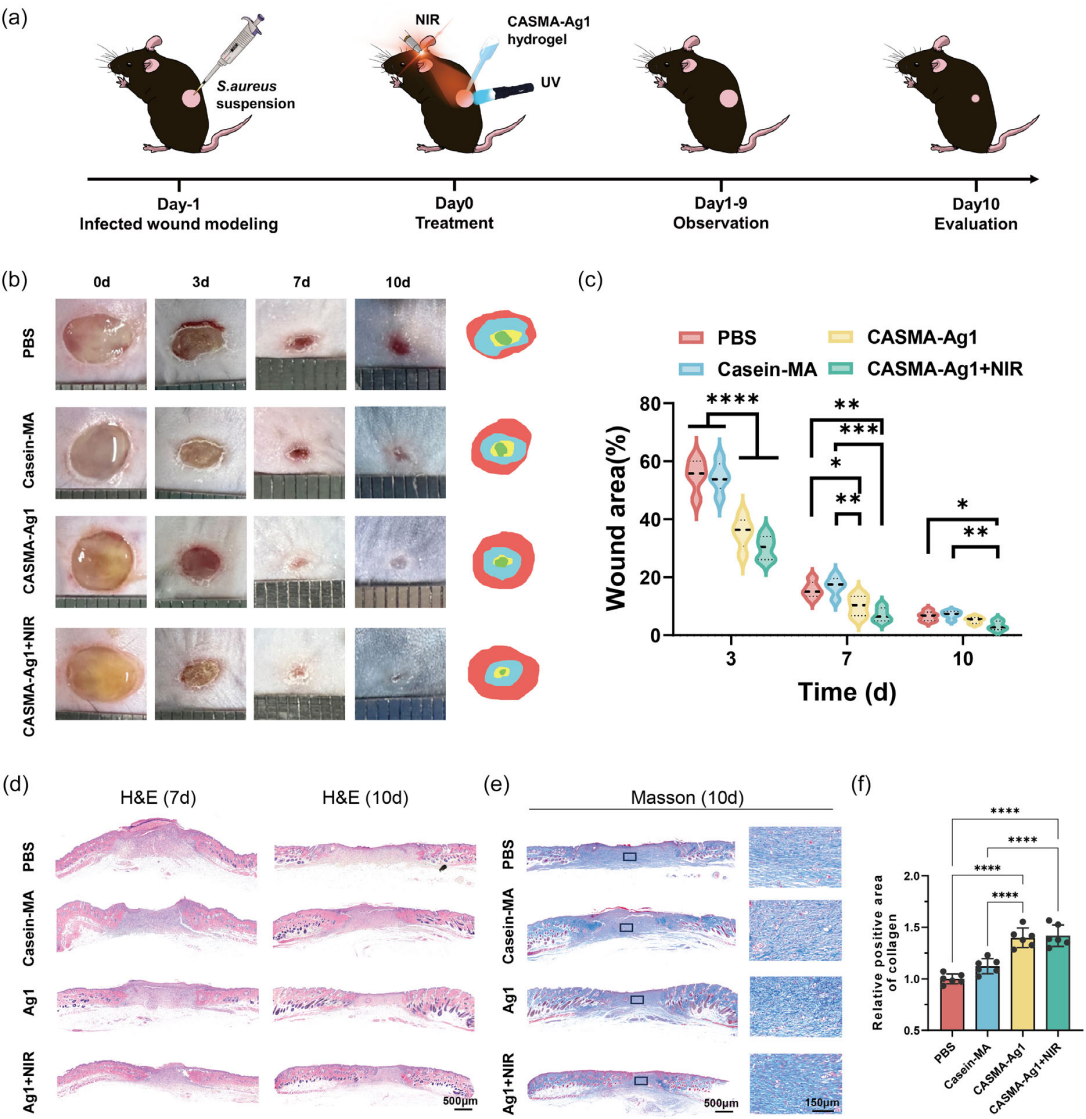
CASMA‐Ag1 hydrogel promotes healing of the infected skin wounds in mice. a) Schematic diagram illustrating the treatment schedule for C57BL/6 mice. b) Representative images of infected wounds at various time points following different treatments. c) The variation of wound area over time for each group. (mean ± SD; *P < 0.05, **P < 0.01, ***P < 0.001, and ****P < 0.0001; n = 5). d) H&E staining showing histological changes during the wound healing process. e) Masson staining of the wound tissue on day 10, and f) quantitative analysis of collagen deposition (mean ± SD; ****P < 0.0001; n = 6).

In addition to its effective biocompatibility, the CASMA‐Ag1 hydrogel had significant antioxidative and antibacterial effects; therefore, we evaluated the wound‐healing performance of the CASMA‐Ag1 hydrogel in vivo using a full‐thickness wound model in infected mice. After infection with *S. aureus* for 24 h, the animals were randomly assigned to four groups and treated with PBS (control), casein‐MA, CASMA‐Ag1, or CASMA‐Ag1 + NIR hydrogel (**Figure** **6**a). The wound tissue fluid was collected on the second day and evaluated using a coating plate (Figure 5g). The number of bacterial colonies in the CASMA‐Ag1 group was significantly lower than that in the PBS and casein‐MA groups. The 405 nm light irradiation used for photoactivated gelation and biomineralization also did not affect the growth of *S. aureus* (Figure S12, Supporting Information). CASMA‐Ag1 + NIR exhibited the highest antimicrobial activity in vivo (Figure 5h), indicating the excellent photothermal antibacterial effect of the hydrogel.

The healing process of infected wounds in mice treated with various formulations (PBS (control), casein‐MA, CASMA‐Ag1, and CASMA‐Ag1 + NIR) was documented by photomonitoring (Figure 6b,c). Throughout the course of the entire treatment, the CASMA‐Ag1 and CASMA‐Ag1 + NIR groups had significantly higher wound closure rates than the other groups. On day 10 posttreatment, wounds treated with CASMA‐Ag1 + NIR achieved a 96.7 ± 1.6% coverage with newly regenerated skin, whereas wounds in the other groups remained overtly visible. The synergistic effect of the NIR photothermal response and intrinsic antimicrobial properties of CASMA‐Ag1 augmented its antimicrobial efficacy and effectively targeted and eradicated pathogens, thus, accelerating the wound healing process. Furthermore, the casein hydrogels used in this study showed decent tissue binding and repair, as reported previously,^[^ ^32^ ^]^ which also facilitated wound healing compared to the control group. Scabs were formed and fell off before healing in all groups. To demonstrate the fate of the CASMA‐Ag1 hydrogel more clearly, the fluorescently labeled CASMA‐Ag1 hydrogel was gelled in situ at the wound site, and the state of the hydrogel was recorded using a fluorescence stereomicroscope at different time points (Figure S13, Supporting Information). The fluorescently labeled hydrogel fell off on about the 7th day.

Pathological staining was used to facilitate a more nuanced analysis of wound healing (Figure 6d). H&E staining on day 7 revealed substantial inflammatory cell infiltration in the PBS and casein‐MA groups. A reduction in the number of inflammatory cells was observed in CASMA‐Ag1 and CASMA‐Ag1 + NIR hydrogel groups. On postoperative day 10, the fundamental structures of the wound epithelium and dermis were observed in each group. Masson's trichrome staining results revealed that both the CASMA‐Ag1 and CASMA‐Ag1 + NIR hydrogel groups demonstrated denser and more reinforced collagen deposition than the other groups, while the CASMA‐Ag1 + NIR group exhibited the most remarkable wound regeneration. Specifically, the epidermis showed thickening and regularity accompanied by the smallest scar area and notable fibroblast proliferation (Figure 6e,f). Our findings suggest that the antibacterial and antioxidative effects of the developed dressings have a potentially positive influence on wound closure, epidermal resurfacing, and collagen deposition, which collectively offer substantial support for skin wound repair.

### 2.7 CASMA‐Ag1 Hydrogel Facilitated Tissue Development and Immune Regulation During Wound Healing

**Figure 7:**
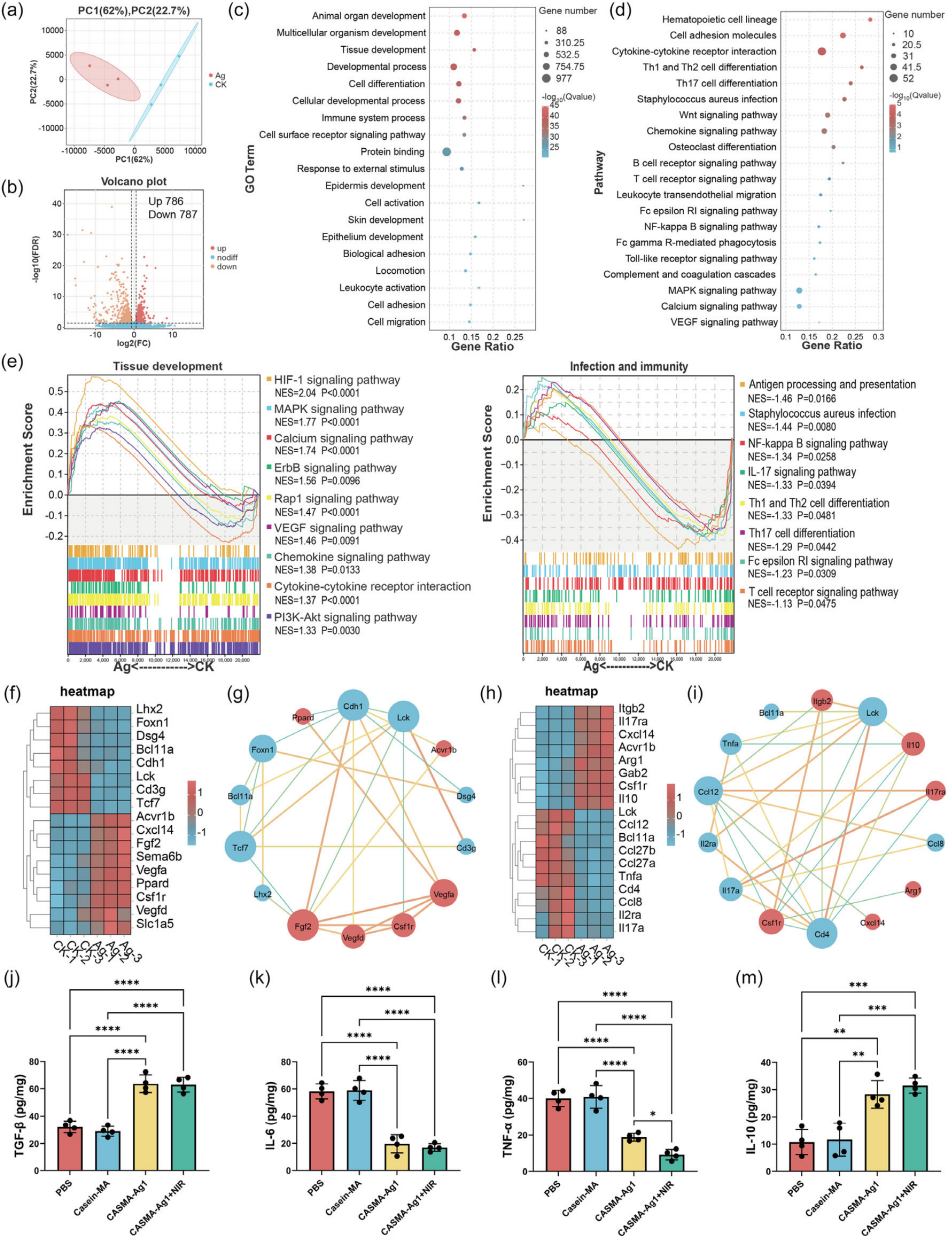
Wound healing mechanisms of the CASMA‐Ag1 + NIR hydrogel. a) PCA of PBS and CASMA‐Ag1 + NIR‐treated groups. b) Volcano plot showing differently expressed genes of PBS versus CASMA‐Ag1 + NIR group. The threshold of log2 (fold change) was 1.5. c) The GO analysis of differently expressed genes in PBS versus CASMA‐Ag1 + NIR. d) The KEGG analysis of differently expressed genes in PBS versus CASMA‐Ag1 + NIR. e) GSEA of differentially expressed signaling pathways involved in tissue development, macrophage polarization, and T cell differentiation following CASMA‐Ag1 + NIR hydrogel treatment. f) Heatmap and g) gene–gene interaction network of significantly differentially expressed genes associated with tissue development after CASMA‐Ag1 + NIR hydrogel treatment. h) Heatmap and i) gene–gene interaction network of significantly differentially expressed genes associated with the immune system process after CASMA‐Ag1 + NIR hydrogel treatment. j–m) ELISA quantification of cytokines in wound tissues: (j) TGF‐*β*, (k) IL‐6, (l) TNF‐α, and (m) IL‐10 (mean ± SD; *P < 0.05, **P < 0.01, ***P < 0.001, and ****P < 0.0001; n = 4).

The potential role of a sequentially photoactivated hydrogel (CASMA‐Ag1 + NIR) in healing infected wounds was investigated by RNA sequencing of mouse wound tissues on the 7th day following treatment with the hydrogel. Principal component analysis (PCA) pointed to a difference in the transcriptomic landscape between the PBS and CASMA‐Ag1 + NIR groups (**Figure** **7**a). A volcano plot of the PBS versus CASMA‐Ag1 + NIR group showed 786 upregulated and 787 downregulated genes (Figure 7b). Gene ontology (GO) analysis indicated that the sequentially photoactivated hydrogel substantially affected the pathways related to organ and tissue development, cell growth, and immune processes (Figure 7c). Kyoto Encyclopedia of Genes and Genomes (KEGG) pathway enrichment analysis revealed that the differentially expressed genes were significantly involved in pathways associated with immune and inflammatory regulation, including T cell differentiation, *S. aureus* infection, B cell and T cell receptor signaling pathways, and leukocyte transendothelial migration (Figure 7d). We also performed gene set enrichment analysis (GSEA) of tissue development and inflammation‐related processes (Figure 7e). Tissue development‐related gene sets (HIF‐1 signaling pathway, VEGF signaling pathway, chemokine signaling pathway, and cytokine–cytokine receptor interaction) were upregulated in the CASMA‐Ag1 + NIR group. For the infection and immunity sets, the enrichment scores of antigen processing and presentation, *S. aureus* infection, NF‐κB signaling pathway, Th1 and Th2 cell differentiation, and Th17 cell differentiation were negative, indicating the antibacterial and antiinflammatory properties of the CASMA‐Ag1 hydrogel. We further performed a heatmap analysis of the gene set related to tissue development and immune regulation and performed a gene–gene interaction network analysis of the differentially expressed genes with the most significantly expressed genes. Genes associated with tissue development, such as VEGFa, PPARd, FGF2, and CSF1r, were significantly upregulated in the CASMA‐Ag1 + NIR group (Figure 7f,g). In contrast, many important genes associated with immunity and inflammation, including proinflammatory cytokines TNF‐a, CCL27, CCL12, IL17a, CD4, IL2ra, and LCK, were markedly downregulated in the CASMA‐Ag1 + NIR group relative to the PBS group (Figure 7h,i). LCK and CD4 are key regulatory nodes of the T‐cell receptor signaling pathway, and downregulation of CD4 and LCK expression affects T‐cell activation and signaling.^[^ ^57^, ^58^ ^]^ ARG1 and IL‐10, known M2 macrophage markers,^[^ ^59^ ^]^ were highly expressed in the CASMA‐Ag1 + NIR group (Figure 7h,i). In addition to defending against pathogens, M2 macrophages clear apoptotic cells, mitigate inflammation, and promote wound healing.^[^ ^60^ ^]^

RNA sequencing analysis suggested that the CASMA‐Ag1 + NIR hydrogel impregnated with AgNPs and resveratrol effectively eliminated bacteria and concurrently diminished oxidative stress, thereby alleviating inflammatory responses and aiding tissue repair.^[^ ^61^, ^62^ ^]^ To further validate the effect of CASMA‐Ag1 on variations in tissue development and inflammatory factors, we quantified the amount of relative cytokines in wound tissues by enzyme‐linked immunosorbent assay (ELISA) assay: Transforming growth factor‐beta (TGF‐β) is a multifunctional growth factor that exert pleiotropic effects on wound healing by regulating cell proliferation and migration, differentiation, extracellular matrix production, and immune modulation.^[^ ^63^ ^]^ Tumor Necrosis Factor‐alpha (TNF‐α) and interleukin 6 (IL‐6) are proinflammatory factors, and interleukin 10 (IL‐10) is an antiinflammatory factor. We observed the upregulation of TGF‐*β* and IL‐10, the downregulation of TNF‐α and IL‐6 in the CASMA‐Ag1 and CASMA‐Ag1 + NIR groups in comparison to the PBS and casein‐MA groups, with the CASMA‐Ag1 + NIR group demonstrating the most pronounced differences from the control groups (Figure 7j−m). Immunohistochemical staining of IL‐6 and TNF‐α in wound tissue sections was performed to study the inflammatory response of different groups. As shown in Figure S14, Supporting Information, a significant amount of yellow or brown IL‐6/TNF‐α positive areas were present in the PBS and casein treatment groups, which were markedly reduced in the CASMA‐Ag1 group and the CASMA‐Ag1 + NIR group. The antiinflammatory effects observed in the CASMA‐Ag1 + NIR group were mainly attributed to the combined antimicrobial effects of AgNPs and photothermal therapy, as well as the inherent antioxidant activity of resveratrol. Collectively, these actions not only alleviate inflammation but also foster the transition to the later stages of wound healing, thereby enhancing overall tissue regeneration. This conclusion is supported by previous studies that highlighted the multifaceted benefits of integrated therapeutic approaches.^[^ ^64^ ^]^ Altogether, our results highlight the significant antibacterial, antiinflammatory, and wound‐healing effects of our hydrogel dressings in vivo, with the sequentially photoactivated group showing particularly promising results.

## 3 Conclusion

In summary, we developed a sequential photoactivatable antibacterial CASMA‐Ag hydrogel with an efficient in situ one‐step UV‐induced biomineralization strategy using casein, resveratrol, and a photosensitive free radical initiator. Cytocompatibility and hemolysis assays demonstrated the excellent biocompatibility of the CASMA‐Ag hydrogel, which is important for wound healing and tissue regeneration. Owing to the favorable photothermal characteristics and intrinsic bactericidal properties of the AgNPs, the hydrogel effectively eradicated both gram‐negative and gram‐positive bacteria. The incorporation of resveratrol not only accelerated in situ synthesis of AgNPs but also provided antioxidant and antiinflammatory properties to the hydrogel, resulting in a significant decrease in the level of ROS in the cells. In mouse models, the CASMA‐Ag hydrogel with sequential radiation demonstrated the ability to eradicate bacteria in vivo, reduce inflammation, and promote wound healing. In the future, we may translate these findings into large animal studies and clinical applications with the ultimate goal of developing a multifunctional wound dressing capable of addressing complex healing challenges. In conclusion, the sequential photoactivatable in situ biomineralization strategy is poised to play a pivotal role in antiinfective wound management and has broad translational prospects.

## 4 Experimental Section

4.1

4.1.1

##### Biomineralization with Diffident Protein

AgNO_3_ was added to a 10% protein solution at a final concentration of 1 mM. 100 μL of each solution was transferred to 96‐well plates and irradiated under 100 mW cm^−2^ and 405 nm UV irradiation for 5 min. Then, the 96‐well plate was scanned at a wavelength of 410 nm using a Spark multifunctional microplate reader (TECAN, Swiss Confederation) to observe AgNP formation.

##### Preparation of CASMA‐Ag Hydrogels

Casein‐MA (0.2 g) was synthesized (as detailed in the supporting information) and dissolved in 1.93 mL of PBS, LAP solution was added (2%, 50 μL), and then Res (100 mg/mL, 10 μL), and various concentrations of AgNO_3_ (0, 100, 200, 300 mM, 10 μL) solution. The solution was irradiated at 100 mW cm^−2^ and 405 nm UV to form hydrogels and AgNPs, respectively. The hydrogels were denoted as CASMA‐Ag0, CASMA‐Ag0.5, CASMA‐Ag1, and CASMA‐Ag1.5.

##### AgNP Adsorption Spectrum

Scanned at wavelengths of 300–700 nm using a Spark multifunctional microplate reader (TECAN, Swiss Confederation).

##### XRD

Crystallization of AgNPs in the hydrogel was assessed by XRD (D8 ADVANCE, Bruker, Germany) in reflection mode with a range of 2θ =10°–80°.

##### TEM

Size and morphology of the AgNPs in the hydrogel were determined by TEM (JEM 2100 F, JEOL, Japan) at a working voltage of 200 kV using a heated powder (producer, brand, etc.). All hydrogel samples (CASMA‐Ag0.5, CASMA‐Ag1, and CASMA‐Ag1.5) were hydrolyzed using protease K to release AgNPs. The precipitate was retained by centrifugation (12 000 *g* min^−1^, 20 min) and washed with ultrapure water three times.

##### Particle Size Distribution (PSD)

PSD was determined by dynamic light scattering using a Zetasizer ZCEC instrument (Malvern Instruments Ltd, UK) with temperature control (25 °C), which was repeated 10 times for each sample.

##### XPS

Elemental content in the hydrogel was determined by XPS (Thermo Scientific K‐Alpha, USA) equipped with a 72 W and 12 kV Al Kα ray source. Wide‐scanning spectra were measured at a passing energy of 150.00 eV and a step size of 1.00 eV. High‐resolution energy spectra were collected at an energy of 50.00 eV and a step size of 0.1 eV. The spectra were fitted and analyzed using Thermo Avantage software v5.948.

##### SEM

Hydrogels were flash‐frozen in liquid nitrogen, dried in a freeze‐drier, sprayed with platinum, and observed using a field emission SEM (SU8010, Hitachi, Japan) equipped with an EDS. Elemental analysis of CASMA‐Ag1 was performed using EDS.

##### Photothermal Experiments Under 808 nm NIR

Hydrogel samples (200 μL) were placed into a 48‐well plate under an 808 nm NIR laser (3 W cm^−2^, 10 min) with a laser module (TZ808AD8000‐F100, Anford, China). Simultaneously, an infrared imaging device (Testo 868, testoAG, Germany) was used to monitor the temperature changes and obtain photothermal images.

##### Rheological Property

Rheological properties of hydrogel samples were assessed at 37 °C using a rheometer (MCR302, Anton Paar, Austria) equipped with a Peltier element for temperature control and a generator. The CASMA‐Ag solutions were placed between the plates at 37 °C to fill the gap (0.1 mm). Under 20 mW cm^−2^, 405 nm UV irradiation, time sweep oscillatory measurements were performed at 50 Hz and 1% strain. The point at which the storage modulus (G′) and loss modulus (G′′) intersect was considered the gelation point, and the point at which the elastic modulus reached a plateau was considered complete crosslinking.

##### Swelling Test

The swelling test was performed as in previous research.^[^ ^32^ ^]^ The specific process is described in the Supporting Information.

##### Stability of the AgNPs in Hydrogel

Hydrogels were covered with aluminum foil and incubated at 37 °C. At time points of 0, 10, and 20 days, representative batches were withdrawn for UV–vis and TEM measurements.

##### Biocompatibility of Hydrogels

Cell biocompatibility and biodegradation were assessed as previously described.^[^ ^32^ ^]^ The specific process is described in the Supporting Information.

##### Hemolysis Evaluation of Hydrogels

Healthy mice were used to obtain “whole” blood samples through venous blood collection or retro‐orbital orbitally. Blood was collected in anticoagulant‐containing tubes with sodium citrate, shaken well, and centrifuged at 3000 rpm for 15 min to obtain blood cells. The extracts were prepared by CASMA‐Ag hydrogels in PBS at an extraction rate of 200 mg mL^−1^ for 12 h at 37 °C. The different extracts, ultrapure water (ddH20), and PBS solution were mixed with 20 μL of blood cells each. The mixture was incubated at 37 °C for 4 h, centrifuged at 3000 rpm for 15 min, and photographed. Absorbance of the supernatant was measured at 542 nm using an enzyme marker.

##### In Vitro Antioxidant Activity

Antioxidant properties of the hydrogels were measured using an ABTS [2,2′ ‐azino‐bis (3‐ethylbenzothiazoline‐6‐sulfonic acid)] assay. The extracts were prepared in PBS at an extraction rate of 200 mg/ml for 12 h at 37 °C. A Total Antioxidant Capacity Assay Kit was used to assess the antioxidant activity of the extracts using the ABTS kit (Beyotime, China). The assay was performed after the incubation of ABTS with an oxidant to produce ABTS+, which has a relatively stable blue‐green color measured at 734 nm. The color suppression was compared with that of Trolox. The assay results are expressed as Trolox equivalents (mM).

##### Intracellular ROS (H_*2*_ *O*_*2*_ *) depletion*

L929 cells were cultured in hydrogel extracts with 200 μM H_2_O_2_ for 24 h. Then, cells were washed with PBS, incubated with the ROS fluorescence probe H2DCFDA (HY‐D0940, MCE, USA; 5 μM) for 30 min, and refreshed with PBS, and photographed immediately. Semiquantitative analysis of intracellular ROS was performed by calculating the fluorescence intensity.

##### Intracellular O_*2*_ *evaluation*

L929 cells were cultured in hydrogel extracts with 200 μM H_2_O_2_ for 24 h. An O_2_ probe Ru(dpp)_3_Cl_2_ (MX4826, MKBio, China) was added into the medium at a concentration of 5 μM in the last 4 h. The cells were washed with PBS and immediately photographed using a fluorescence microscope (Leica DMI8). The fluorescence of Ru(dpp)_3_Cl_2_ was quenched in the presence of oxygen. Thus, a semiquantitative analysis of intracellular O_2_ was performed by calculating the reduction in fluorescence.

##### In Vitro Antibacterial Effect

To evaluate the antibacterial effect with or without NIR irradiation, 200 μL of the hydrogels was added in a 48‐well plate and 50 μL containing a bacterial suspension of *E. coli* (ATCC 8099) or *S. aureus* (ATCC 25 923) diluted with PBS and was incubated with or without NIR (808 nm, 3 W cm^−2^, 10 min). After a 12 h incubation at 37 °C, the bacteria were resuspended and diluted 10^−4^ times in PBS and then plated on a Luria–Bertani (LB) agar for 24 h. The colony forming units per milliliter (CFU/mL) were calculated for each group and compared with the control. The bactericidal effects of the hydrogels were verified using the LIVE/DEAD BacLightTM Bacterial Viability Kit (Sigma‐Aldrich, USA). Briefly, bacterial suspensions from the different hydrogel treatments were stained with propidium iodide (PI) and SYTO 9 fluorescent dye for 15 min in the dark. The bacteria were then washed three times with physiological saline to remove excess dye. For each group, live and dead bacterial cells were observed and visualized using an inverted fluorescence microscope (IX73; Olympus, Japan). The numbers of live and dead cells were quantified using ImageJ software.

##### Mouse S. Aureus‐Infected Cutaneous Wound Model

The performance of the CASMA‐Ag1 hydrogel in wound healing was tested using an *S. aureus*‐infected cutaneous wound model. All animal experiments were approved by the Experimental Animal Management Committee of Dr. Can Biotechnology (Zhejiang) Co., Ltd. (Reference Number: 2024DRK0029). Briefly, male C57BL/6 mice (6 weeks old, 19‐21.0 g) were purchased from Shanghai Slac Laboratory Animal Co. Ltd. After anesthetization with pentobarbital (30 mg kg^−1^ body weight) and removal of the dorsal hair, 6 mm diameter full‐thickness skin round wounds were created, and the suspension of *S. aureus* (10 μL, 10^8^ CFU mL^−1^) was immediately dropped onto the wounds to establish the full‐thickness *S. aureus*‐infected wound model and randomly divided into four groups: PBS, casein‐MA, CASMA‐Ag1, and CASMA‐Ag1 + NIR. After 24 h, the control group was treated with PBS, and the other groups were completely covered with the corresponding hydrogels under 100 mW cm^−2^ and 405 nm UV irradiation for 5 min. Then, the wounds in the last group were irradiated with the NIR laser (3 W cm^−2^) for 10 min. After 2 d of treatment, 1 μL of tissue fluids from wounds with different treatments were collected and diluted with PBS by 10^4^ times. 100 μL diluent was uniformly coated on an LB medium plate, and the medium plate was placed in a biochemical incubator at 37 °C for inverted culture. After 24 h, the number of bacterial cells in the culture medium was determined. The CFU was calculated using the following formula.

CFU=100×growing colonies

Photographs were taken to monitor the wound‐healing process of the mice. The wound areas were measured using the ImageJ software.

##### Histology

After 3, 7, and 10 days of treatment, the mice were euthanized, and the wound skin tissue was collected. All collected samples were fixed in a 4% paraformaldehyde solution and embedded in paraffin to prepare tissue slides with a thickness of 5 μm, which were stained with hematoxylin and eosin (H&E), Masson trichrome staining, and for histological observation using a Leica DM3000 microscope (Germany). In addition, immunohistochemical staining (TNF‐α and IL‐6) of wound tissue on day 7 was performed. Collagen content (collagen%) was quantified by analyzing the proportion of aniline blue‐stained area (S_Blue_) in the total tissue area (S_Tissue_) using the following equation: Pixel areas of S_Blue_ and S_Tissue_ were segregated from the original images using the color threshold function of the ImageJ software.

$$Collagen\%=\frac{S_{Blue}}{S_{Tissue}}\times 100\%$$

##### Whole Genome RNA Sequencing

The wound tissues on the 7th day were collected and washed with PBS, and stored at –80 °C before sequencing. After total RNA extraction using a TRIzol reagent kit (Invitrogen, Carlsbad, CA, USA), eukaryotic mRNA was enriched using oligo (dT) beads. Eukaryotic mRNA sequencing was performed using an Illumina Novaseq6000 (Gene Denovo Biotechnology Co., Guangzhou, China). The NEBNext Ultra RNA Library Prep Kit for Illumina (NEB #7530; New England Biolabs, Ipswich, MA, USA) was used to construct the cDNA library. The following processes were performed: total RNA extraction, mRNA enrichment, mRNA fragmentation, random hexamer‐primed cDNA synthesis, size selection, PCR amplification, and Illumina sequencing.

##### ELISA of Cytokines

Wound tissue was collected on day 7 and homogenized to retain the supernatant. The total protein content in the supernatant was determined using a bicinchoninic acid (BCA) Protein Assay Kit (Beyotime, China). The inflammation in the wound was assessed using a commercial ELISA kit (FANKEWEI, China).

##### Statistical Analysis

All experiments were performed in triplicate. Quantitative values are shown as the mean ± standard deviation of at least three independent experiments. The experimental data were assessed for normality using the Shapiro–Wilk test before analysis. Comparisons between two groups were made using a two‐tailed unpaired Student's *t*‐test, while one‐way ANOVA was applied to analyze differences among three or more groups, followed by a Tukey post hoc test. All analyses were completed using GraphPad Prism 9.0 and Origin 2022. Statistical significance is indicated by *P < 0.05, **P < 0.01, ***P < 0.001, and ****P < 0.0001.

## Conflict of Interest

The authors declare no conflict of interest.

## Author Contributions

**Qinchao Zhu** and **Xuhao Zhou**: investigation, methodology, writing—original draft preparation. **Zhidan Wang**: investigation, validation. **Daxi Ren**: supervision, resources, writing—reviewing and editing, funding acquisition. **Tanchen Ren**: formal analysis, supervision, resources, funding acquisition, writing—reviewing and editing.

## Supporting information

Supplementary Material

## Data Availability

The data that support the findings of this study are available from the corresponding author upon reasonable request.
