# Supplementary material for: Milk‐Derived Injectable Wound Dressing with Sequential Photoactivatable Antibacterial Property through In Situ Biomineralization
Source: Small Sci. 2025 Jun 23;5(9):2500026. doi: 10.1002/smsc.202500026 (PMC12412551; doi:10.1002/smsc.202500026)
Supplement: Supplementary file 1 — Supplementary Material [file SMSC-5-2500026-s001.pdf]

Supporting Information

Milk Derived Injectable Wound Dressing with Sequential Photoactivatable Anti-bacteria through In-situ Biomineralization

Qinchao Zhu[1,#], Xuhao Zhou[1,2,#], Zhidan Wang[1], Daxi Ren[1,3,*], Tanchen Ren[1,2,4*]

[1] *Department of Cardiology, The Second Affiliated Hospital, School of Medicine, Institute of Dairy Science, College of Animal Sciences, Zhejiang University, Hangzhou, China*

[2] *State Key Laboratory of Transvascular Implantation Devices, Heart Regeneration and Repair Key Laboratory of Zhejiang Province, Hangzhou, China*

[3] *College of Animal Sciences, Xinjiang Agricultural University, Urumqi, China*

[4] *Transvascular Implantation Devices Research Institute, Hangzhou, China*

[†]Qinchao Zhu and Xuhao Zhou contributed equally to this work

*Corresponding Authors：

Tanchen Ren: rentanchen120@zju.edu.cn, Daxi Ren: dxren@zju.edu.cn.

Materials and methods

Methacrylation of Casein: 100 g casein (Fonterra, Shanghai, China) was dissolved in 1000 mL of 0.1 M NaOH solution at 25−30 °C, and then, 25 mL methacrylic anhydride (purity 94%, with 0.2% topanol) (Macklin Biochemical Co., Ltd., Shanghai, China) was added to the casein solution. The mixture was stirred for 24 h at 25−30 °C, and the pH of the mixture was maintained at 8 with 5 M NaOH. Subsequently, refer to the previous ultrafiltration method used in the laboratory[1,2], the resulting solution was ultrafiltered at 25 °C with an HMTECH-UF1812 bench-top ultrafiltration system (Huamo Technology Co. Ltd., Hangzhou, China). Finally, methacrylated casein (Casein-MA) solutions were frozen at −80 °C for 12 h and freeze-dried for 24 h. Lyophilized CASMA powder was stored at −20 °C for further use.

1H NMR Method: NMR spectra were collected using a Bruker AVANCE NEO 600 MHz NMR spectrometers equipped with a cryoprobe. The samples were dissolved at a concentration of 20 mg in 1mL of H_2O/D_2O 90/10 (v/v) for NMR characterization. Degree of substitution

(DoS) was described by the following formula:

$$DoS(\%) = \left[1 - \frac{A(lysine\ methene\ of\ Casein - Ma)}{A(lysine\ methere\ of\ casein)}\right] \times 100\%$$

where A (lysine residues of casein-MA) represents the signal intensity of lysine methene in the methacrylated casein. A (lysine residue of casein) represents the signal intensity of lysine methene in the water-soluble casein.

Ninhydrin Assay: The Casein and casein-MA were formulated at a concentration of 5 mg/mL. The protein solutions (1mL) were mixed with 0.5 mL 1% w/v ninhydrin solution. PBS (0.5 mL) was added to the mixture and incubated in boiling water for 15 min for a color reaction. The resulting solutions were then photographed.

Circular Dichroism Spectroscopy: Circular dichroism (CD) spectra were collected on a Chirascan V100 instrument within a spectral range of 185-260 nm with an integrating sphere attachment. For each test, n=3.

Fourier Transform Infrared Spectra: Casein and Casein-MA were scanned by a FTIR spectrophotometer (Termo Scientifc Nicolet iS20, Termo Electron Co., Waltham,

MA, USA), at 32 times per second, from 4000 to 400 cm^{-1}.

3D Printing: The 10% (w/v) Casein-MA solution was poured into the resin tank of a digital light printer. a bottom-up DLP printer, consisting of an ink tank, a Z-direction motion platform, and a 405 nm UV projector with 1280 × 720 dpi resolution and 17.6 W cm^{-2} light intensity. A computer-aided design (CAD) model of the target-printed construct was designed in SolidWorks and sliced into a series of 2D images using the Creation Workshop software. The designed 3D structure was sliced with a layer height of 100 µm for printing, and the printing time for each layer was set as 50 s.

Swelling Test of CASMA-Ag Hydrogel: The initial weight of the hydrogel (8 mm diameter and 6 mm height) was accurately measured after fabrication. Then, the hydrogel samples were immersed in 50 mL PBS buffer (pH=7.4) at 25 °C for 48 h. After specified time intervals, the swollen hydrogels were weighed by gently removing excess water using filter paper. The swelling ratio is defined as follows:

$$swelling\ ratio = \frac{mi - m0}{m0}$$

where mi and m0 are the weights of the swollen and initial samples, respectively.

Cell Biocompatibility of Hydrogels: Hydrogel extracts, prepared from various bulk formulations, were immersed in DMEM medium supplemented with 10% FBS at a concentration of 200 mg/ml for a period of 12 hours at 37 °C. L929 mouse fibroblasts were then seeded into 96-well cell culture plates (5×10^3 cells per well) and cultured for 12 h. Then, the culture medium was replaced with 100 µL of extract, with fresh medium maintained in the control group. After a 24-hour incubation, cell viability was determined using the Cell Counting Kit-8 (Yeasen Biotech, China) and Live/Dead staining kit (Yeasen Biotech, China), according to the manufacturer's protocols. For the CCK8 assay, the absorbance at 450 nm was quantified using a microplate reader (Tecan M200 PRO, Tecan Company, Switzerland) after a 2-hour interval. For live/dead fluorescence staining, images were obtained using a fluorescence microscope (Leica DMI8, Germany) and subsequently analyzed using ImageJ software to calculate the proportion of viable cells.

In vivo degradation: CASMA-Ag1 hydrogel precursor solution was exposed to

blue light in a 1 mL syringe to form cylindrical hydrogels. The hydrogel samples were cut into 0.1 g pieces. Male C57BL/6N mice (20 g) were anesthetized using pentobarbital. After hair on the back was removed and disinfected, the skin was incised symmetrically on both sides of the midline. Crosslinked hydrogels were implanted subcutaneously, and the wounds were sutured. At 7, 14, 21, and 28 days after implantation, the animals were sacrificed, and the hydrogels and surrounding tissues were excised and photographed. The skin and internal organs were stained with HE. At 28 days, mouse blood was biochemically analyzed (ALT, AST, ALP, ALB, UREA, and CREA).

Ultraviolet (405 nm) sterilization ability: Briefly, male C57BL/6 mice (6 weeks old, 19-21.0 g) were purchased from Shanghai Slac Laboratory Animal Co. Ltd. and were randomly divided into two groups: PBS and PBS + UV. After anesthetization with pentobarbital (30 mg kg^{-1} body weight) and removing the dorsal hair, 6 mm diameter full thickness skin round wounds were created and the suspension of *S. aureus* (10 μL, 10^8 CFU mL^{-1}) was immediately dropped onto the wounds to establish the full-thickness *S. aureus*-infected wound model. After 24 h, group BL was treated with PBS and PBS+UV groups were with 100 mW cm^{-2} and 405 nm UV irradiation for 5 min. After 2 d of treatment, 1 μL tissue fluids from wounds with different treatment were collected and diluted with PBS by 10^4 times. 100 μL diluent was uniformly coated on LB medium plate, and the medium plate was placed in a biochemical incubator at 37 °C for inverted culture. After 24 h, the number of bacterial cells in the culture medium was determined. The CFU were calculated using the following formula:

CFU = 100 × growing colonies

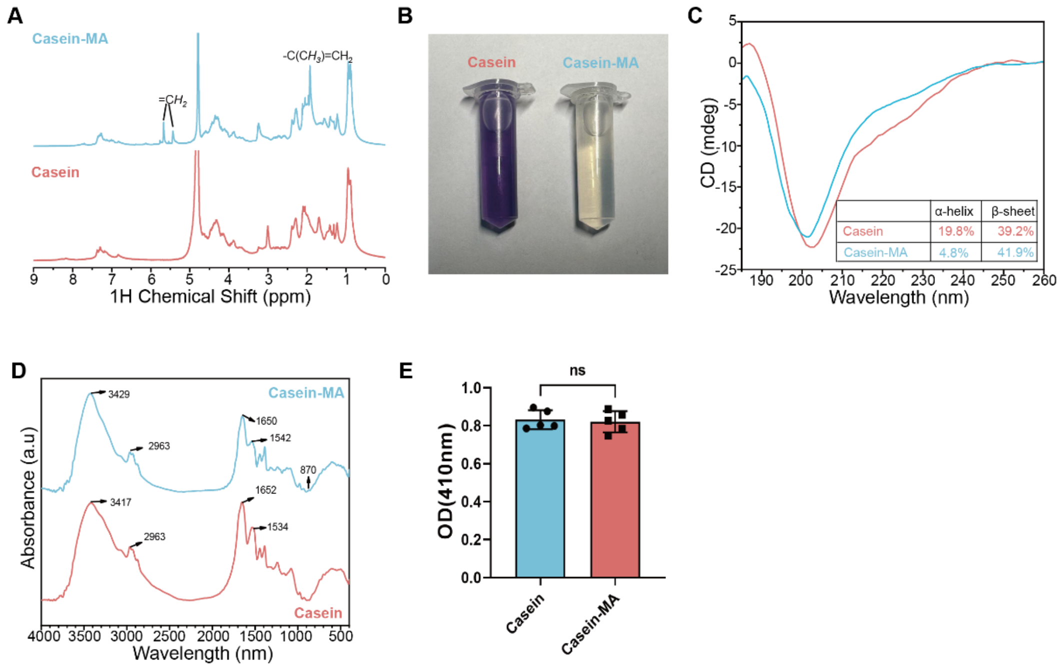

Figure S1. Methacrylation of casein. A) Solid-state NMR spectra of casein and Casein-MA. B) Representative images showing chromogenic results of casein and Casein-MA with the ninhydrin test. C) Solid-state circular dichroism spectra of casein and Casein-MA. D) FT-IR spectra of casein and Casein-MA. E) The OD (410 nm) value after UV-activated AgNPs biomineralization with casein and Casein-MA. (mean ± SD; ns, $P > 0.05$; n = 5).

Methacrylic anhydride (MA) was grafted onto the casein backbone to synthesize Casein-MA via methacrylate substitution of the primary amines on casein. According to the acylation mechanism, the methacryloyl group is added to the nitrogen or oxygen atoms of lysine, histidine, and serine residues, and the byproduct is methacrylic acid [3]. The DoS of the modified casein was determined by a ninhydrin assay and 1H NMR spectroscopy [4]. After purification by dialysis, the characteristic peak resonances of the methacrylate vinyl group (δ = 5.4 ppm) and the methyl group of Casein-MA (δ = 2.8 ppm) were observed from the 1HNMR spectra (Figure S1A). The DoS of Casein-MA was 99.8% obtained via lysine methene signal (δ= 2.92 ppm) intensity. The ninhydrin assay revealed that Casein-MA has a relatively weaker purple color than casein (Figure S1B. CD spectra showed a slight change in the secondary structure of casein, and the overall α-helix content decreased after methacrylate (Figure S1C). This was caused by the methacrylation of some residues in the original casein or a change in the protein environment during the modification process. The

FTIR results demonstrated that casein and Casein-MA were not significantly different. The strong absorption peaks at 1650, 1652 (amide I band), 1534, and 1542 cm^{-1} (amide II band) were the stretching vibration peaks of C=O and N–H in amide bond, respectively (Figure S1D). The peak located at 2963 cm^{-1} belonged to C–H stretching vibration peak. A total of 3429 cm^{-1} and 3417 cm^{-1} absorption peaks were overlapping peaks of O–H and N–H stretch vibration. The overlapping peaks of the modified casein were narrower because the amino and hydroxyl groups were partially replaced, indicating that methacryloyl groups were introduced into casein. In addition, the absorption peak at 870 cm^{-1} was the stretching vibration peak of C–H on terminal olefin, while the casein without anhydride modifcation was weaker, which also demonstrated that modified casein was successfully synthesized. Methacrylization did not affect the biomineralization of casein (Figure S1F).

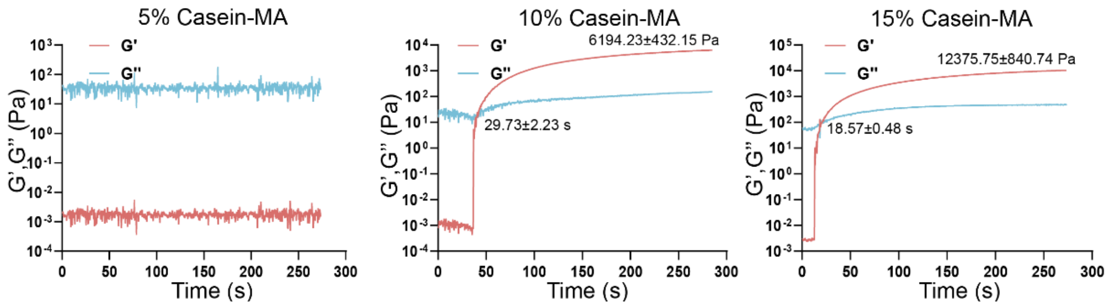

Figure S2. Rheology analysis of hydrogels with different Casein-MA concentration (5%,10%,15%).

The rheological properties of Casein-MA hydrogels were monitored during photocuring to evaluate the effect of Casein-MA concentration on the gelation time and storage modulus (G') (Figure S2). After exposure to blue light (405 nm, 1 W cm^{-2}), the storage modulus began to increase to a certain point until it gradually stabilized. The final storage modulus after complete gelation was 6194.23±432.15 Pa for 10% Casein-MA, and 12375.75±840.74 Pa for 15% Casein-MA.

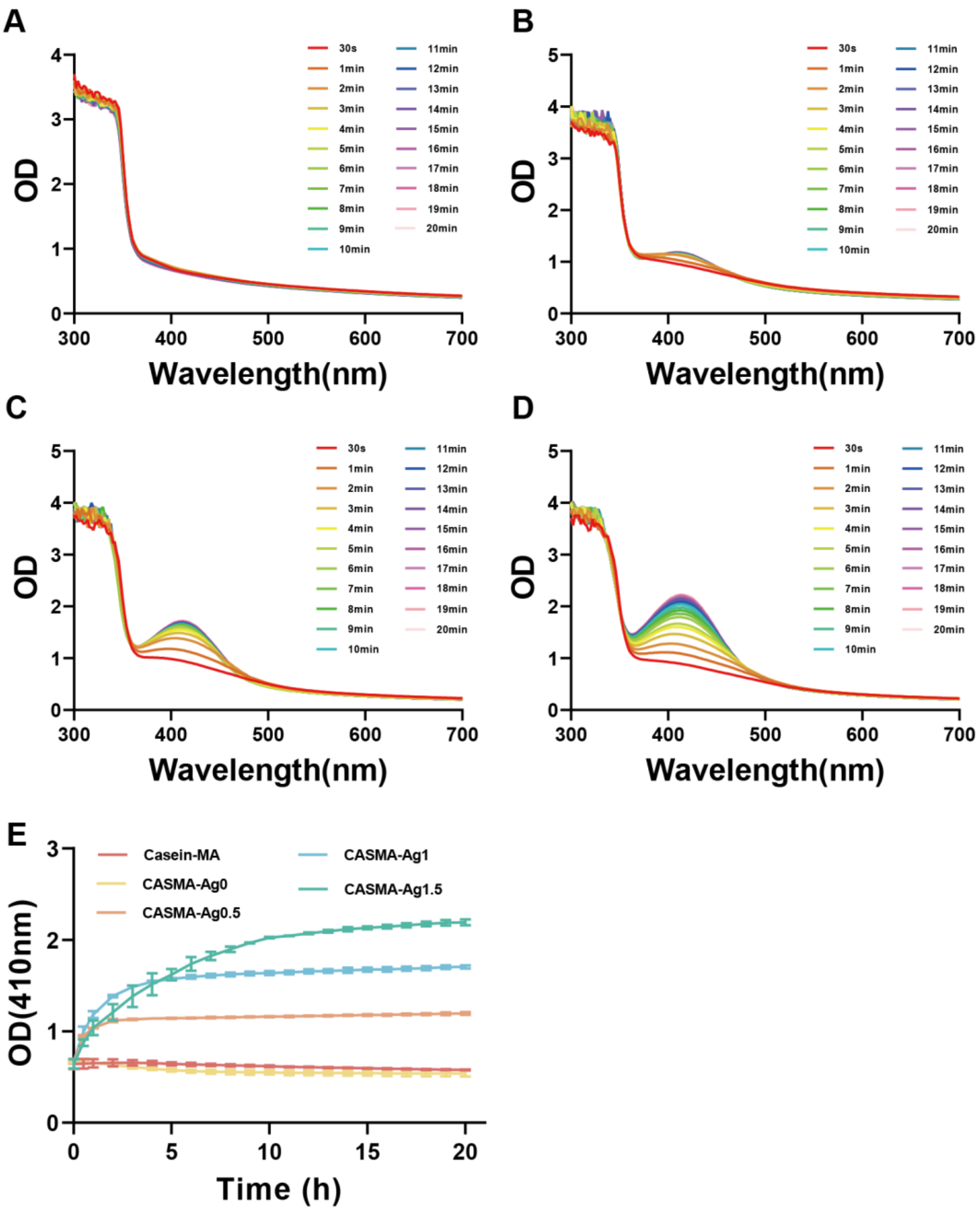

Figure S3. Characterization of nano-silver generation with UV-Vis absorption spectra. A-D) The UV-Vis absorption spectra of CASMA-Ag0(A), CASMA-Ag0.5(B), CASMA-Ag1(C), and CASMA-Ag1.5(D) with different UV- irradiation time. E) The OD (410 nm) value of different hydrogel samples with the increase of UV-irradiation time.

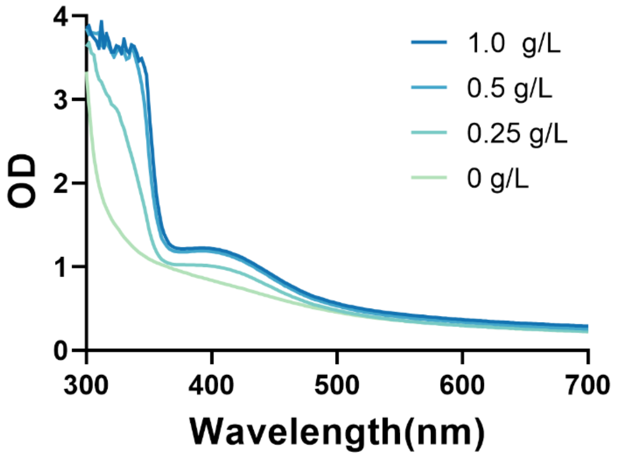

Figure S4. UV–vis spectra of the AgNPs hydrogel with different Res concentration.

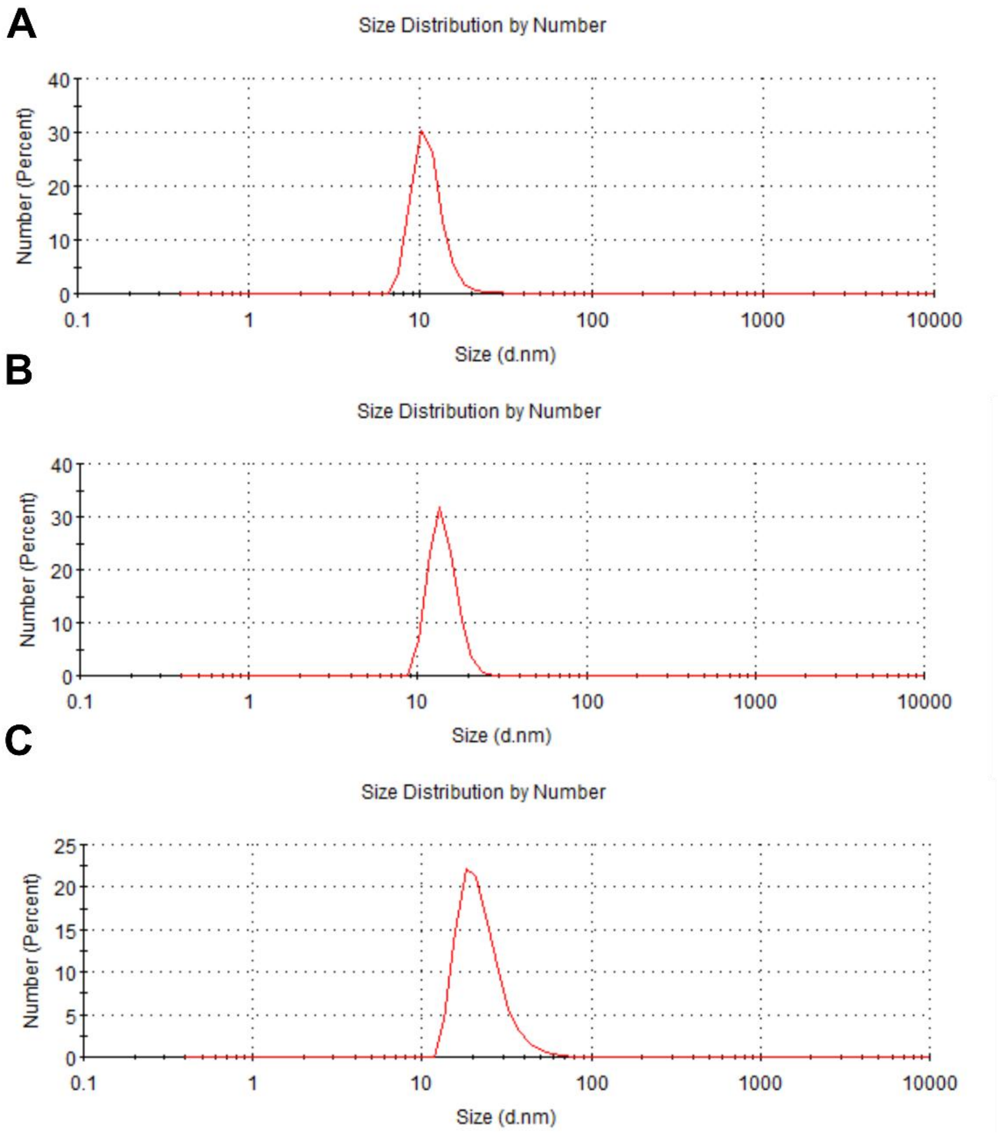

Figure S5. Size distribution of AgNPs in (A) CASMA-Ag0.5, (B) CASMA-Ag1, and (C) CASMA-Ag1.5 by DLS.

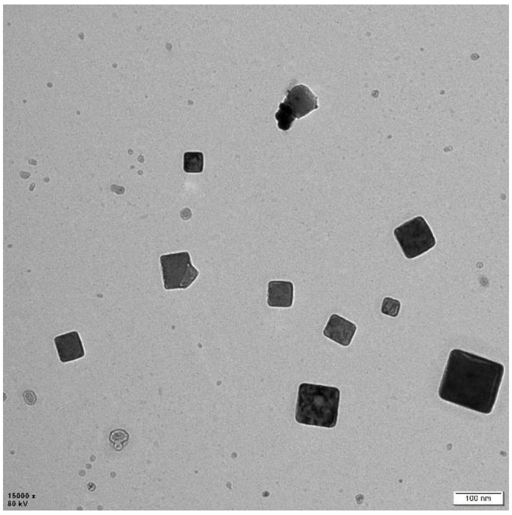

Figure S6. Transmission electron micrographs of AgNPs in 15% Casein-MA.

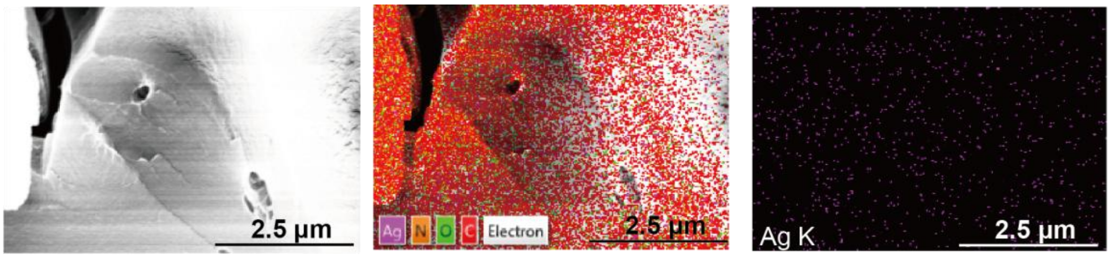

Figure S7. Elemental mapping images of C, O, N, and Ag of CASMA-Ag1.

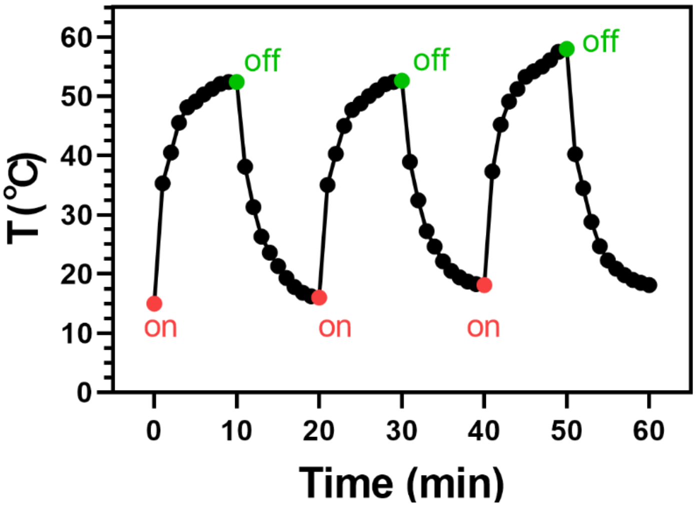

Figure S8. Temperature changes of the CASMA-Ag1 hydrogel during three laser irradiation on/off cycles.

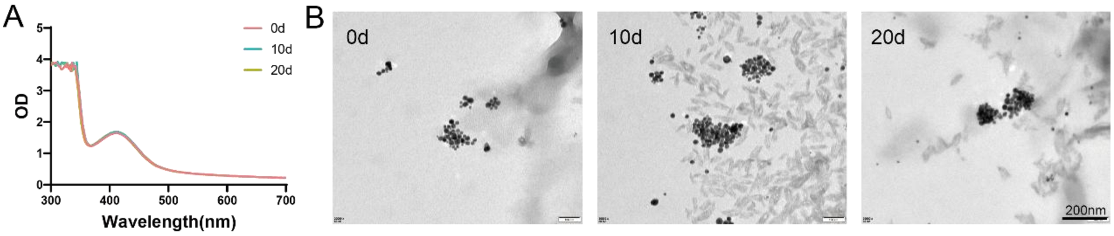

Figure S9. A) UV-Vis spectroscopy of CASMA-Ag1 hydrogel at day 0, day 10, and day 20. B) The TEM measurements of AgNPs in CASMA-Ag1 hydrogel at day 0, day 10, and day 20.

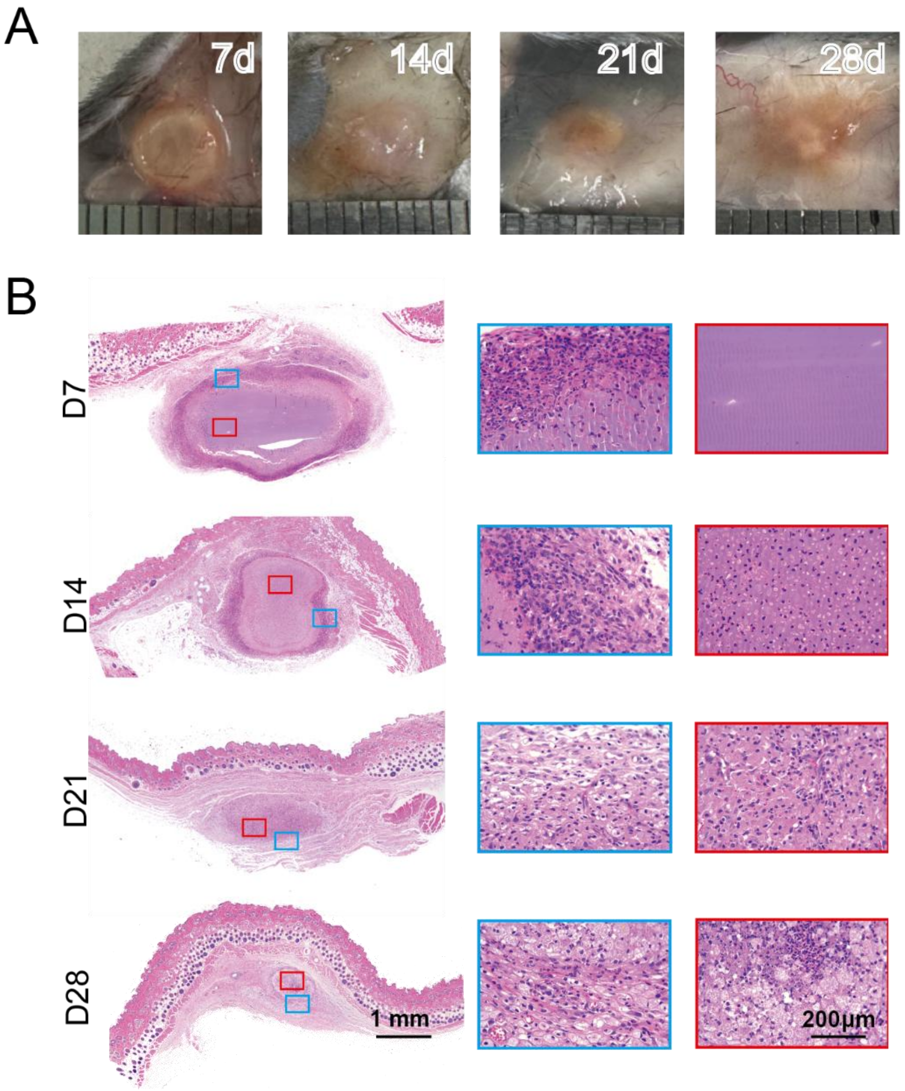

Figure S10. *In vivo* biodegradation of CASMA-Ag1 hydrogels. A) Gross appearance of CASMA-Ag1 after implantation. B) H&E staining images of CASMA-Ag1 hydrogels 7, 14, 21 and 28 days after implantation.

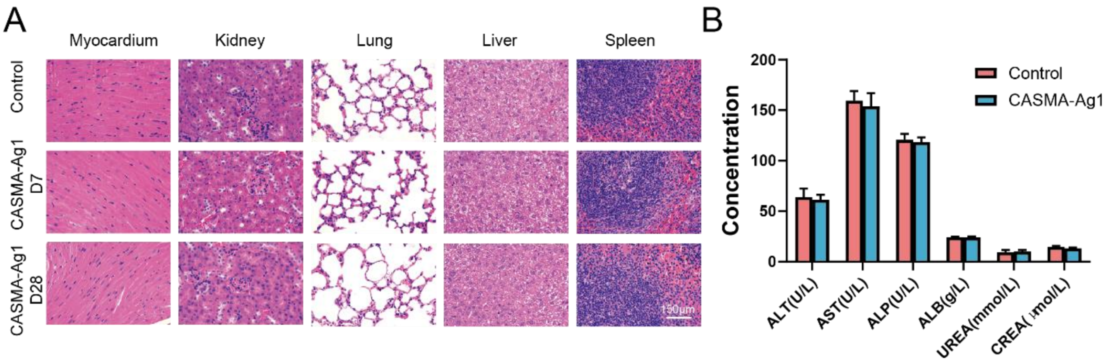

Figure S11. A) H&E staining of major organs (heart, liver, spleen, lung and kidney) of mice at 7 and 28 days after CASMA-Ag1 subcutaneous implantation. B) Biochemical analysis of mouse blood at 28 days after CASMA-Ag1 subcutaneous implantation (mean ± SD; n = 3)..

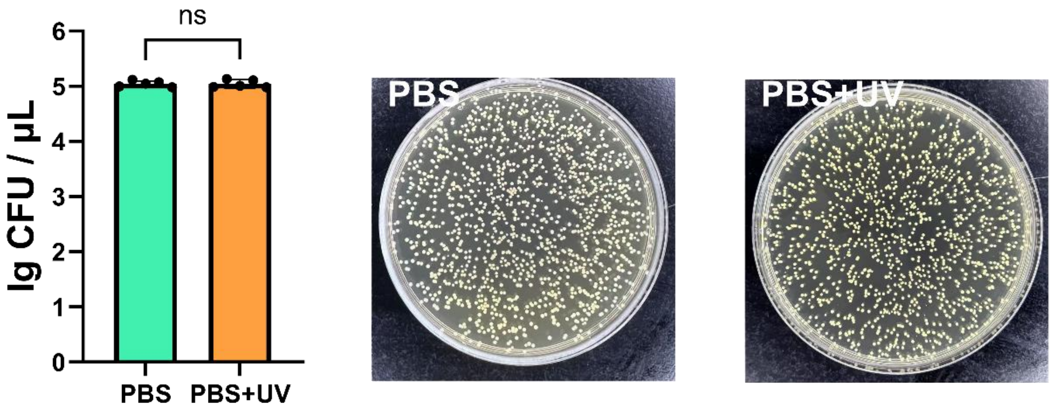

Figure S12. CFU analyzed of *S.aureus* collected from infected wounds treated by PBS with or without 405 nm light irradiation (mean ± SD; ns, $P > 0.05$; n = 5).

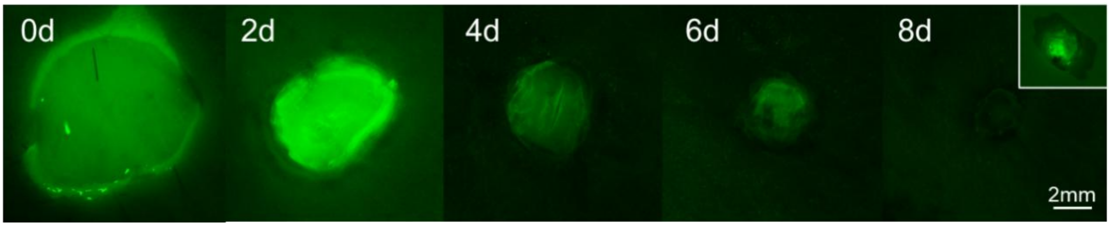

Figure S13. Fluorescence images of fluorescent-labeled CASMA-Ag1 hydrogel at the wound healing site at day 2, day 4, day 6 and day 8.

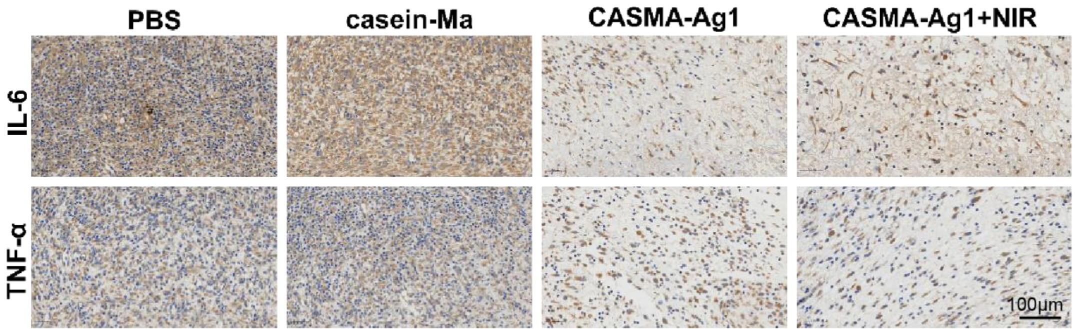

Figure S14. Immunohistochemistry of wound tissue with different treatments at day 7.

References

[1] Y. Huang, Q. Zhu, Y. Zhu, T. G. Valencak, Y. Han, T. Ren, C. Guo, D. Ren, *ACS Omega* **2023**, acsomega.3c05793.

[2] Z. Hu, W. Cao, L. Shen, Z. Sun, K. Yu, Q. Zhu, T. Ren, L. Zhang, H. Zheng, C. Gao, Y. He, C. Guo, Y. Zhu, D. Ren, *ACS Appl. Mater. Interfaces* **2022**, *14*, 28501.

[3] J. G. Werner, S. Nawar, A. A. Solovev, D. A. Weitz, *Macromolecules* **2018**, *51*, 5798.

[4] S. S. Sali, M. L. Gould, M. Qasim, M. A. Ali, *J. Mater. Chem. B* **2021**, *9*, 1557.
